# A unified view of density-based methods for semi-supervised clustering and classification

**DOI:** 10.1007/s10618-019-00651-1

**Published:** 2020-07-27

**Authors:** Jadson Castro Gertrudes, Arthur Zimek, Jörg Sander, Ricardo J. G. B. Campello

**Affiliations:** 1 SCC/ICMC/USP, University of São Paulo, Avenue Trabalhador São-carlense, 400 - Center, São Carlos, SP 13566-590 Brazil; 2 IMADA, University of Southern Denmark, Campusvej 55, 5230 Odense M, Denmark; 3 Department of Computing Science, University of Alberta 1-001 CCIS, Edmonton, AB T6G-2E9 Canada; 4 School of Mathematical and Physical Sciences, University of Newcastle, University Drive, Callaghan, NSW 2308 Australia

**Keywords:** Semi-supervised classification, Semi-supervised clustering, Density-based clustering

## Abstract

Semi-supervised learning is drawing increasing attention in the era of big data, as the gap between the abundance of cheap, automatically collected unlabeled data and the scarcity of labeled data that are laborious and expensive to obtain is dramatically increasing. In this paper, we first introduce a unified view of density-based clustering algorithms. We then build upon this view and bridge the areas of semi-supervised clustering and classification under a common umbrella of density-based techniques. We show that there are close relations between density-based clustering algorithms and the graph-based approach for transductive classification. These relations are then used as a basis for a new framework for semi-supervised classification based on building-blocks from density-based clustering. This framework is not only efficient and effective, but it is also statistically sound. In addition, we generalize the core algorithm in our framework, HDBSCAN*, so that it can also perform semi-supervised clustering by directly taking advantage of any fraction of labeled data that may be available. Experimental results on a large collection of datasets show the advantages of the proposed approach both for semi-supervised classification as well as for semi-supervised clustering.

## Introduction

Semi-supervised learning algorithms tackle cases where a relatively small amount of labeled data yet a large amount of unlabeled data is available for training (Chapelle et al. 2006; Zhu and Goldberg 2009). We find examples of semi-supervised learning scenarios in various fields, such as email filtering, sound/speech recognition, text/webpage classification, and compound discovery, just to mention a few. For instance, in areas such as biology, chemistry, and medicine, domain experts and laboratory analyses may be required to label observations, thus only a small collection of labeled data can usually be afforded, which may not be representative enough for supervised learning to be applied (Batista et al. 2016).

Typically, semi-supervised learning algorithms are based on extensions of either supervised or unsupervised algorithms by including additional information in the form originally handled by the other learning paradigm. For instance, in semi-supervised *clustering*, a collection of labeled observations can be used to guide the (otherwise unsupervised) search for clustering solutions that better meet users’ prior expectations. Labels in clustering only indicate whether observations are expected to be part of the same cluster or different clusters, there is no one-to-one association between the unique labels known to the user and the possible categories to be discovered in the data. In semi-supervised *classification*, the classes are known in advance, and unlabeled observations are used in addition to the labeled ones to improve on (the otherwise supervised) classification performance.

Semi-supervised learning can be categorized into *inductive* and *transductive* learning. Inductive learning uses both labeled and unlabeled training data to generate a model able to predict the labels for the unlabeled training data as well as for future data to be labeled. Transductive learning predicts the labels of the unlabeled training data only (from which a model may *optionally* be derived afterwards, if prediction of new, unseen data objects is required). In transductive *classification*, which is the focus of the first part of this paper, a large amount of unlabeled objects can be classified based on a small fraction of labeled objects, which is not representative enough to successfully train a classifier in a traditional, fully supervised way. Label-based semi-supervised*clustering*, which is the focus of the second part of this paper, is intrinsically transductive in its nature. The main difference from transductive classification is that in clustering not necessarily all possible categories and their labels are known in advance, so unlabeled objects may be assigned newly discovered labels that are not present in the original training set.

In the case of semi-supervised classification, unlabeled data can help improve classification performance when there is a good match between the problem structure and the model’s assumptions: “*...there’s no free lunch. Bad matching of problem structure with model assumption can lead to degradation in classifier performance*” (Zhu 2005). Different models of semi-supervised classification exist, relying on different model assumptions (Zhu 2005). One of the major paradigms, clustering-based models, follows the well-known *cluster assumption* of semi-supervised classification:

### Assumption 1

(*Cluster assumption*; Chapelle et al. 2006) If points are in the same cluster, they are likely to be of the same class.

This assumption is quite general and broad in scope as there are many possible interpretations of “cluster”, under different clustering paradigms. An important, statistically sound paradigm is *density-based clustering* (Kriegel et al. 2011), where clusters are defined as high-density data regions separated by low-density regions. Under this paradigm, Assumption 1 is closely related to another common assumption in semi-supervised classification:

### Assumption 2

(*Smoothness assumption*; Chapelle et al. 2006) The label function is smoother in high-density than in low-density regions...If two points in a high-density region are close, then so should be their outputs (labels)...If, on the other hand, they are separated by a low-density region, then their outputs need not be close.

According to Chapelle et al. (2006), in the context of classification and from a density-based clustering perspective, Assumptions 1 and 2 are equivalent to each other and can be read as “*The decision boundary should lie in a low-density region (low density separation)*”. In spite of the obvious connections between these two areas, however, the use of density-based clustering for semi-supervised classification has been surprisingly overlooked in the literature. Many methods focus instead on the use of graphs (as opposed to clusters) to model the notions of locality and connectivity of the data (de Sousa et al. 2013). Such graph-based methods mostly rely on the following model assumption:

### Assumption 3

(*Graph assumption*; Zhu and Goldberg 2009) Class labels are “smooth” with respect to the graph, so that they vary slowly, i.e., if two points are connected by a strong edge, their labels tend to be the same.

In this paper we show that there is a strong relation between density-based clustering methods and the graph-based approach for transductive classification, by first establishing formal relationships between a number of key unsupervised and semi-supervised clustering algorithms under a unified view of density-based clustering, then establishing the links and interpretations of these algorithms from the perspective of graph theory. Taking advantage of such a unified view, we then firstly introduce a framework of density-based clustering for semi-supervised classification that brings the three assumptions above (namely cluster, smoothness, and graph) under a common umbrella. In this context, we make the following initial contributions: (a) our framework extends the state-of-the-art density-based hierarchical clustering algorithm HDBSCAN* (Campello et al. 2015), originally proposed as an unsupervised or (constraint-based) semi-supervised clustering algorithm, to perform transductive *classification* from a small collection of pre-labeled data objects; (b) we show that, in the context of transductive classification, other well-known density-based algorithms for semi-supervised clustering can also be derived as particular cases, with the advantage that our framework eliminates possible order-dependency and graph re-computation issues of these algorithms, while being simpler and easier to interpret; and (c) by combining building blocks from different algorithms, a number of novel variants follow naturally from our framework, which, to the best of our knowledge, have never been tried before.

We published the aforementioned contributions in a preliminary conference paper (Gertrudes et al. 2018). The current paper is an extension of this preliminary publication that expands our unified view of density-based methods from the semi-supervised classification scenario to the label-based semi-supervised *clustering* scenario, where labels for certain categories may be missing in the training set. As a novel contribution in this context, we extend HDBSCAN*, which plays a central role in our unified view and framework for density-based classification, to also perform semi-supervised clustering from a collection of pre-labeled data objects, rather than instance-level pairwise constraints (as currently supported by the algorithm). The direct use of labels can be shown to be both simpler and more effective. To that end, a new collection of experiments focused on clustering has also been included as extended material in this paper, in addition to the classification experiments from our preliminary publication (Gertrudes et al. 2018).

The remainder of this paper is organized as follows: in Sect. 2 we discuss related work that is close to our approach. In Sect. 3 we present our unified view of density-based clustering algorithms that bridges between the areas of semi-supervised clustering and classification. In Sect. 4 we introduce our framework for density-based semi-supervised classification. In Sect. 5 we present our newly proposed strategy to perform label-based semi-supervised clustering. In Sects 6 and 7 we discuss our experiments and results, respectively. Finally, in Sect. 8 we conclude the paper and discuss some future work.

## Related work

In the context of semi-supervised classification, different categories of algorithms have been described in the literature (Zhu 2005). Closer to our work are the *clustering-based* and the *graph-based* approaches. Graph-based algorithms construct a graph with vertices from both labeled and unlabeled objects. Generally, neighboring vertices are connected by edges such that edge weights are proportional to some measure of local connectivity strength (de Sousa et al. 2013). Once the neighborhood graph is built, labels can be somehow transfered from labeled to unlabeled objects, e.g., using Markov chain propagation techniques (Szummer and Jaakkola 2002) or regularized methods based on the graph Laplacian (Zhao et al. 2006). The Laplacian SVM (LapSVM) (Belkin et al. 2006) is related to the latter category and is a state-of-the-art algorithm in the semi-supervised classification literature.

A well-known strategy for label propagation in graph-based semi-supervised classification is the use of a so-called harmonic function. In this context, a harmonic function is a function that has the same values as the labels on the labeled data, and satisfies the weighted average property on the unlabeled data, i.e., the value assigned to each unlabeled object is the weighted average of the values of its neighbors. In a binary classification problem, the class labels are coded, e.g., as $\{-1,+1\}$, and these values are assigned to the vertices corresponding to the labeled objects. Each remaining (unlabeled) object has its value determined as the average of the values of its adjacent vertices in the neighborhood graph, weighted by the corresponding edge weights. The resulting real values, which allow for different physical and probabilistic interpretations, can be discretized back into $\{-1,+1\}$ to achieve the final transductive classification. A classic algorithm that follows this type of approach is the Gaussian Field Harmonic Function (GFHF) (Zhu et al. 2003).

To address multi-class semi-supervised classification, Liu and Chang (2009) formulated a constrained label propagation problem by incorporating class priors, leading to a simple closed-form solution. The algorithm, called Robust Multi-Class Graph Transduction (RMGT), is an extension of the GFHF algorithm, which can be viewed as a constrained optimization problem using a graph Laplacian as smoothness measure (de Sousa 2015). Both RMGT and GFHF, as well as the previously mentioned LapSVM algorithm, are used as baseline for comparisons in our experimental evaluation.

In contrast to graph-based methods, clustering-based algorithms for semi-supervised classification perform label transduction based on the clustering structure of the data, rather than by using an explicit graph (Zhu and Goldberg 2009). However, since certain clustering techniques are implicitly or explicitly built upon graphs and related algorithms, there are connections between these two different paradigms of semi-supervised learning, which are investigated in this paper. Of particular interest in our context are density-based clustering methods (Kriegel et al. 2011), which are popular in the data mining field as a statistically sound approach that has also been used for semi-supervised classification, and has also been shown to have strong connections with elements from graph theory (Campello et al. 2015). Two noticeable algorithms in this context are HISSCLU (Böhm and Plant 2008) and Semi-Supervised DBSCAN (SSDBSCAN) (Lelis and Sander 2009), both of which are built upon notions inherited from two classic, widely used unsupervised density-based clustering algorithms, namely, OPTICS (Ankerst et al. 1999) and DBSCAN (Ester et al. 1996).

SSDBSCAN (Lelis and Sander 2009), which has more recently also been extended to the active learning scenario (Li et al. 2014), was in principle proposed as a semi-supervised clustering method, which does not necessarily label all objects (as it would normally be expected in transductive classification), but rather leave certain objects unlabeled as noise, as usual (and meaningful) in density-based *clustering* applications. Despite this, the algorithm explicitly relies on a *classification* assumption:

### Assumption 4

(*Classification assumption*) There is at least one (possibly more) labeled object from each class.

Unlike SSDBSCAN, HISSCLU (Böhm and Plant 2008) already includes an extended label-propagation scheme that assigns a label from the training set to every unlabeled object in the database. It further differs from SSDBSCAN in that it also includes a preprocessing mechanism to widen the gap between nearby classes by stretching distances between objects around class boundaries. This mechanism allows HISSCLU to expand different class labels even within clusters that are formed by more than one class (i.e., clusters of objects that are density connected but not pure in their labels). SSDBSCAN, in contrast, makes the *label consistency* assumption:

### Assumption 5

(*Label consistency assumption*) “Label consistency requires different labels to belong to different clusters; under this assumption, a single class can still have multiple modes [clusters or sub-clusters]; in other words, objects in different clusters can have the same label, only in a single cluster the labels have to be the same.” (Lelis and Sander 2009)

From this perspective, SSDBSCAN relies more strictly than HISSCLU on the *clustering assumption of semi-supervised classification* (Assumption 1). Unlike SSDBSCAN, which was originally proposed for the semi-supervised clustering task, HISSCLU can perform both semi-supervised classification and clustering. Both remain state-of-the-art algorithms in the density-based literature, so they are also used as baseline for comparisons in our experimental evaluation.

Apart from SSDBSCAN and HISSCLU, very few algorithms exist in the realm of semi-supervised density-based clustering. Ruiz et al. (2007, 2010) proposed C-DBSCAN, which is a modified version of DBSCAN designed to cope with instance-level constraints. However, C-DBSCAN has the same limitation as DBSCAN in that it uses a single, critical global density threshold determined by two user-defined parameters. In addition, the algorithm enforces constraints in a hard sense, i.e., clusters under cannot-link constraints are not allowed to be formed and different clusters under must-link constraints are forced to be merged. Hence, while satisfying the user-provided constraints, the algorithm violates the implicit assumptions behind the clustering model adopted, namely, the definitions of density connectivity and density-based clusters.

An algorithm of particular interest that does not suffer from any of the above limitations is HDBSCAN* (Campello et al. 2013a, 2015). Originally, HDBSCAN* was proposed as a method for unsupervised or (constraint-guided) semi-supervised density-based clustering. In this paper, HDBSCAN* is extended in two different ways: first, it is extended to also perform semi-supervised classification via label propagation; second, it is extended to perform semi-supervised clustering directly from labels, rather than instance-level pairwise constraints.

In the following section we discuss in more detail fundamental concepts and ideas underpinning the algorithms DBSCAN, OPTICS, SSDBSCAN, HISSCLU, and HDBSCAN*, while establishing the links between these algorithms as well as their connections with graph theory, which will be subsequently required to understand our proposed unified approach for density-based semi-supervised clustering and classification.

## A unified view of density-based clustering algorithms

In this document, we adopt the following notations: $\mathbf {X} = \{\mathbf {x}_{1}, \mathbf {x}_{2}, \ldots , \mathbf {x}_{n}\}$ is a dataset with *n* data objects, $\mathbf {x}_{i}$. Some of the algorithms described in this paper assume that data objects are points in a *d*-dimensional Euclidean space, i.e., $\mathbf {x}_{i} \in \mathbb {R}^{d}$ is a *d*-dimensional feature vector with real-valued coordinates ($\mathbf {x}_{i} = [x_{i1} \cdots x_{id}]^{{\textsf {T}}}$). Others do not make any assumptions about features and only require a measure of dissimilarity between pairs of data objects, $d(\mathbf {x}_{i},\mathbf {x}_{j})$, in order to operate. This dissimilarity is assumed to be a distance but not necessarily a metric. $\mathbf {X}_{L} \subset \mathbf {X}$ is a subset of the data objects for which class labels are available, and ${{\,\mathrm{class}\,}}(\mathbf {x}_{i})$ is the class label of object $\mathbf {x}_{i} \in \mathbf {X}_{L}$. The subset of unlabeled objects is denoted by $\mathbf {X}_{U}$, such that $\mathbf {X}_{L} \cup \mathbf {X}_{U} = \mathbf {X}$ and $\mathbf {X}_{U} = \mathbf {X}~\backslash ~ \mathbf {X}_{L}$.

### DBSCAN*, DBSCAN, and OPTICS

A number of concepts used later in this work refer back to ideas and definitions from DBSCAN (Ester et al. 1996), OPTICS (Ankerst et al. 1999), and related algorithms. We start describing DBSCAN*, which is a simplified version of DBSCAN defined in terms of *core* and *noise objects* only (Campello et al. 2013a):

#### Definition 1

(*Core and noise*) An object $\mathbf {x}$ is called a core object w.r.t. $\epsilon \in \mathbb {R}_{\ge 0}$ and $m_{\text {pts}} \in \mathbb {N}_{> 0}$ if its $\epsilon$-neighborhood (a ball of radius $\epsilon$ centered at $\mathbf {x}$) contains at least $m_{\text {pts}}$ many objects, i.e., if $|N_{\epsilon }(\mathbf {x})| \ge m_{\text {pts}}$, where $N_{\epsilon }(\mathbf {x})=\{ \mathbf {x}_{i} \in \mathbf {X} ~|~ d(\mathbf {x}, \mathbf {x}_{i}) \le \epsilon \}$ and $| \cdot |$ stands for set cardinality. An object is called noise if it is not a core object.

#### Definition 2

($\epsilon$*-reachable*) Two core objects $\mathbf {x}_{i}$ and $\mathbf {x}_{j}$ are $\epsilon$-reachable w.r.t. $\epsilon$ and $m_{\text {pts}}$ if $\mathbf {x}_{i} \in N_{\epsilon } (\mathbf {x}_{j})$ and $\mathbf {x}_{j} \in N_{\epsilon } (\mathbf {x}_{i})$.

#### Definition 3

(*Density-connected*) Two core objects $\mathbf {x}_{i}$ and $\mathbf {x}_{j}$ are density-connected w.r.t. $\epsilon$ and $m_{\text {pts}}$ if they are directly or transitively $\epsilon$-reachable.

#### Definition 4

(*Cluster*) A cluster $\mathbf {C}$ w.r.t. $\epsilon$ and $m_{\text {pts}}$ is a non-empty maximal subset of $\,\mathbf {X}$ such that every pair of objects in $\mathbf {C}$ is density-connected.

Like in DBSCAN, two parameters define a density threshold given by a minimum number of objects, $m_{\text {pts}}$, within a ball of radius $\epsilon$ centered at an object $\mathbf {x}$. Clusters are formed only by objects $\mathbf {x}$ satisfying this minimum density threshold (core objects). Two such objects are in the same cluster if and only if they can reach one another directly or through a chain of objects in which every consecutive pair is within each other’s $\epsilon$-neighborhood.

DBSCAN* is not only simpler, but it is also statistically more rigorous than the original DBSCAN, as it strictly conforms with the classic principle of *density-contour clusters* as defined by Hartigan (1975). The original DBSCAN relaxes this principle by allowing some objects below the density threshold, called *border objects*, to be incorporated into clusters. Specifically, a border object in DBSCAN is a non-core object that lies within the $\epsilon$-neighborhood of a core object. For convenience, here we will formalize this notion by using the following definitions adapted from OPTICS (Ankerst et al. 1999):

#### Definition 5

(*Core distance*) The core distance of an object $\mathbf {x}_{i} \in \mathbf {X}$ w.r.t. $m_{\text {pts}}$, $d_{\text {core}}(\mathbf {x}_{i})$, is the distance from $\mathbf {x}_{i}$ to its $m_{\text {pts}}$-nearest neighbor (where the 1st-nearest neighbour is by convention the query object itself, $\mathbf {x}_{i}$, the 2nd-nearest neighbour is thus the next object closest to $\mathbf {x}_{i}$, and so on).

#### Definition 6

(*Reachability distance*) The (asymmetric) reachability distance from an initial object $\mathbf {x}_{i}$ to an end object $\mathbf {x}_{e}$ w.r.t. $m_{\text {pts}}$, $d_{\text {reach}}(\mathbf {x}_{i},\mathbf {x}_{e})$, is the largest of the core distance of $\mathbf {x}_{i}$ and the distance between $\mathbf {x}_{i}$ and $\mathbf {x}_{e}$: $d_{\text {reach}}(\mathbf {x}_{i},\mathbf {x}_{e}) = \max \{d_{\text {core}}(\mathbf {x}_{i}), d(\mathbf {x}_{i},\mathbf {x}_{e})\}$.

From Definition 5, it is clear that the core distance of an object $\mathbf {x} \in \mathbf {X}$ is the minimum value of the radius $\epsilon$ for which $\mathbf {x}$ is a core object (i.e., its density is above the minimum density threshold). By definition, a border object $\mathbf {x}_{e}$ in DBSCAN is not a core object, which means $\epsilon < d_{\text {core}}(\mathbf {x}_{e})$. Also by definition, a border object $\mathbf {x}_{e}$ falls within the $\epsilon$-neighborhood of a core object, say $\mathbf {x}_{i}$, which means $d(\mathbf {x}_{i},\mathbf {x}_{e}) \le \epsilon$ and, since $\mathbf {x}_{i}$ is core, $\epsilon \ge d_{\text {core}}(\mathbf {x}_{i})$. From these inequalities, it follows that $d_{\text {core}}(\mathbf {x}_{e}) > \epsilon \ge \max \{d_{\text {core}}(\mathbf {x}_{i}), d(\mathbf {x}_{i},\mathbf {x}_{e})\}$, and using Definition 6 a border object can then be defined as:^1^

#### Definition 7

(*Border object*) An object $\mathbf {x}_{e} \in \mathbf {X}$ is called a border object w.r.t. $\epsilon$ and $m_{\text {pts}}$ if $d_{\text {core}}(\mathbf {x}_{e}) > \epsilon$ and there exists another object $\mathbf {x}_{i} \in \mathbf {X}$ such that $\epsilon \ge d_{\text {reach}}(\mathbf {x}_{i},\mathbf {x}_{e})$.

The clusters in the original DBSCAN are the same as in DBSCAN*, augmented with their corresponding border objects; all the other objects are labeled as noise by both algorithms. From a graph perspective, it is straightforward to see that the clusters in DBSCAN* (Definition 4) are the connected components of an undirected graph where each core object is represented as a vertex and two vertices are adjacent if and only if the corresponding core objects fall within each other’s $\epsilon$-neighborhood (i.e., iff they are $\epsilon$-reachable—Definition 2). DBSCAN also includes border objects as vertices, each of which is adjacent to a core object. For a border object $\mathbf {x}_{e}$, if there is more than one core object $\mathbf {x}_{i}$ satisfying Definition 7, the original DBSCAN makes $\mathbf {x}_{e}$ adjacent to one of those chosen randomly, but the choice can be made deterministically, e.g., the one that minimizes $d_{\text {reach}}(\mathbf {x}_{i},\mathbf {x}_{e})$ (or $d(\mathbf {x}_{i},\mathbf {x}_{e})$ in case of ties).

Starting from an arbitrary object in the dataset, OPTICS (Ankerst et al. 1999) derives an *ordering* ($\prec$) of the data objects that implicitly encodes all possible DBSCAN solutions for a given value of $m_{\text {pts}}$. The algorithm does not require the radius $\epsilon$ to produce such an ordering, which has the following property: given an object $\mathbf {x}_{q}$, the smallest reachability distance to $\mathbf {x}_{q}$ from any of its preceding objects is no greater than the smallest reachability distance from any of its preceding objects to an object succeeding $\mathbf {x}_{q}$, i.e.,

$$\begin{aligned} \min _{\mathbf {x}_{p}:\, \mathbf {x}_{p} \prec \mathbf {x}_{q}} d_{\text {reach}}(\mathbf {x}_{p},\mathbf {x}_{q}) \le \min _{\begin{array}{c} \mathbf {x}_{o},\mathbf {x}_{r} :\\ \mathbf {x}_{o} \prec \mathbf {x}_{q} \prec \mathbf {x}_{r} \end{array}} d_{\text {reach}}(\mathbf {x}_{o},\mathbf {x}_{r}). \end{aligned}$$

This property is important for two reasons: (a) it ensures that by plotting $\min _{\mathbf {x}_{p}:\, \mathbf {x}_{p} \prec \mathbf {x}_{q}} d_{\text {reach}}(\mathbf {x}_{p},\mathbf {x}_{q})$ for every object $\mathbf {x}_{q}$ in the given order, the so-called OPTICS *reachability plot*, density-based clusters and sub-clusters appear as valleys or “dents” in the plot; and (b) if one wants to set a threshold $\epsilon$, as a horizontal line cutting through the plot, it is straightforward to show that DBSCAN clusters with radius $\epsilon$ correspond essentially to the contiguous subsequences of the ordered points for which the plot is below the threshold.

The OPTICS ordering and reachability plot can be easily achieved by keeping an adaptable priority queue sorted by the smallest reachability distance from an object outside the queue (already processed) to each object inside the queue. At each iteration, the object with the smallest such distance (priority key) is removed from the queue (processed), the reachability distances from that object to the objects inside the queue are computed, and the queue is readjusted accordingly. From a graph perspective, this is algorithmically analogous to Prim’s algorithm to compute a Minimum Spanning Tree (MST), the only difference being that OPTICS operates on a directed graph where each pair of vertices ($\mathbf {x}_{p},\mathbf {x}_{q}$) is connected by a pair of unique edges, one in each direction, whose weights are the corresponding reachability distances, i.e. $d_{\text {reach}}(\mathbf {x}_{p},\mathbf {x}_{q})$ and $d_{\text {reach}}(\mathbf {x}_{q},\mathbf {x}_{p})$. The optional threshold $\epsilon$ to extract DBSCAN clusters corresponds to pruning out from such a complete digraph any edge whose weight is larger than $\epsilon$. The strongly connected components of the resulting digraph (subsets of vertices mutually reachable via directed paths) correspond to the DBSCAN* clusters with radius $\epsilon$, whereas the DBSCAN clusters additionally include vertices that are not part of any of the strongly connected components, but are reachable from those (i.e., the border objects).

### SSDBSCAN

#### Conceptual approach

SSDBSCAN (Lelis and Sander 2009) is a semi-supervised algorithm that performs semi-supervised clustering of an unlabeled dataset $\mathbf {X}_{U} \subset \mathbf {X}$ from a small fraction of labeled data $\mathbf {X}_{L} \subset \mathbf {X}$ using a label expansion engine that is very similar to OPTICS. Unlike OPTICS, however, SSDBSCAN circumvents the unnecessary complications related to border objects and the asymmetric nature of the original reachability distance in Definition 6 by using a symmetric version of it, which has been formally defined as *mutual reachability distance* by Campello et al. (2013a, 2015):

##### Definition 8

(*Mutual reachability distance*) The mutual reachability distance between two objects $\mathbf {x}_{i}$ and $\mathbf {x}_{j}$ in $\mathbf {X}$ w.r.t. $m_{\text {pts}}$ is defined as $d_{\text {mreach}}(\mathbf {x}_{i}, \mathbf {x}_{j}) = \max \{d_{\text {core}}(\mathbf {x}_{i}), d_{\text {core}}(\mathbf {x}_{j}), d(\mathbf {x}_{i},\mathbf {x}_{j})\}$.

The interpretation of this definition plays a fundamental role not only in SSDBSCAN but also more broadly here in our work: the mutual reachability distance is the *smallest value of the radius*$\epsilon$ for which the corresponding pair of objects are still core objects and are $\epsilon$-reachable from each other (Definition 2). For a given $m_{\text {pts}}$, $\epsilon$ establishes a density threshold that is inversely proportional to this radius, and the mutual reachability distance is hence*inversely proportional to the largest density threshold* for which the corresponding pair of objects is directly density-connected according to Definition 3.

Conceptually, SSDBSCAN attempts to solve the following problem: for each unlabeled object, $\mathbf {x}_{i} \in \mathbf {X}_{U}$, the goal is to assign $\mathbf {x}_{i}$ the same label, ${{\,\mathrm{class}\,}}(\mathbf {x}_{j})$, as the object $\mathbf {x}_{j} \in \mathbf {X}_{L}$ that is the “closest” to $\mathbf {x}_{i}$ from a density-connectivity perspective, if such a labeling is possible, without violating the *label consistency* assumption (Assumption 5). This assumption requires that objects with different labels have to reside in disjunct density-based clusters following Definition 4 (w.r.t. the same $m_{\text {pts}}$, but possibly different $\epsilon$ values).

Let us provisionally put the label consistency assumption aside and first focus on the primary goal, namely, what “*closest to*$\mathbf {x}_{i}$*from a density-connectivity perspective*” means. In density-based clustering, this refers to the object that can reach out (or be reached from) $\mathbf {x}_{i}$ through a path along which the lowest density connection is as high as possible (in other words, the *weakest* point in the connection is as strong as possible). Notice that, in light of Definition 8, this is equivalent to the path along which the largest*mutual reachability distance* is as small as possible. From a graph perspective, if objects are vertices and any two vertices are connected by an undirected edge weighted by their mutual reachability distance, the goal is to find the path between the unlabeled vertex $\mathbf {x}_{i}$ in question and a labeled vertex $\mathbf {x}_{j}$ along which the largest edge is minimal. Given $\mathbf {x}_{i}$ and any labeled candidate $\mathbf {x}_{j}$, this corresponds to the classic *minmax* problem in graph theory, whose solution can be proven to be the path between $\mathbf {x}_{i}$ and $\mathbf {x}_{j}$ along the minimum spanning tree (MST) of the graph. Hence, a preliminary approach to label the set of unlabeled objects $\mathbf {X}_{U} \subset \mathbf {X}$ could be the following:

##### Definition 9

(*Label propagation*) Compute the MST of the dataset $\mathbf {X}$ in the transformed space of mutual reachability distances, $\text {MST}_r$, find the largest $\text {MST}_r$ edge connecting each object $\mathbf {x}_{i} \in \mathbf {X}_{U}$ to every object $\mathbf {x}_{j} \in \mathbf {X}_{L}$, and make ${{\,\mathrm{class}\,}}(\mathbf {x}_{i}) = {{\,\mathrm{class}\,}}(\mathbf {x}_{j})$ where $\mathbf {x}_{j}$ is the labeled object for which such a maximum edge is minimal.

**Fig. 1:**
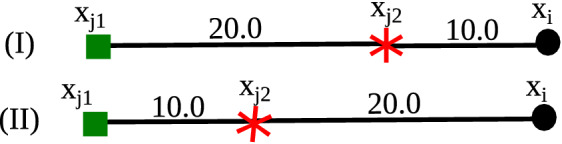
SSDBSCAN label assignment: in case (I), object $\mathbf {x}_{i}$ is assigned to $\mathbf {x}_{j2}$ (red star); in case (II), $\mathbf {x}_{i}$ is left unlabeled as noise (Color figure online)

From the density-based *clustering* perspective, however, there is a problem with this approach. Let us consider two labeled objects, $\mathbf {X}_{L} = \{\mathbf {x}_{j1}, \mathbf {x}_{j2}\}$, such that ${{\,\mathrm{class}\,}}(\mathbf {x}_{j1}) \ne {{\,\mathrm{class}\,}}(\mathbf {x}_{j2})$ and $\mathbf {x}_{j2}$ is on the $\text {MST}_r$ path between $\mathbf {x}_{j1}$ and an unlabeled object $\mathbf {x}_{i} \in \mathbf {X}_{U}$. Now consider two possible scenarios:

- In the first scenario, the largest $\text {MST}_r$ edge on the path between $\mathbf {x}_{j1}$ and $\mathbf {x}_{i}$, say 20, is located on the sub-path between $\mathbf {x}_{j1}$ and the intermediate object, $\mathbf {x}_{j2}$. The largest edge on the path between $\mathbf {x}_{j2}$ and $\mathbf {x}_{i}$ is then smaller than 20, say 10 (Fig. 1-I). In this case, it is safe to make ${{\,\mathrm{class}\,}}(\mathbf {x}_{i}) = {{\,\mathrm{class}\,}}(\mathbf {x}_{j2})$ without violating the label consistency assumption because any threshold $\epsilon \in [10,20)$ can make $\mathbf {x}_{i}$ density-connected to $\mathbf {x}_{j2}$ (and, therefore, part of the same density-based cluster according to Definition 4) but not to $\mathbf {x}_{j1}$.
- In the second scenario, the largest $\text {MST}_r$ edge on the path between $\mathbf {x}_{j1}$ and $\mathbf {x}_{i}$, say 20, is located on the sub-path between $\mathbf {x}_{j2}$ and $\mathbf {x}_{i}$, whereas the largest edge on the path between $\mathbf {x}_{j1}$ and $\mathbf {x}_{j2}$ is, say, 10 again (Fig. 1-II). In this case, the largest edge on the path between $\mathbf {x}_{i}$ and the two labeled objects in question is the same, yet those two labeled objects have different labels. There is no threshold that can keep $\mathbf {x}_{i}$ density-connected to either $\mathbf {x}_{j1}$ or $\mathbf {x}_{j2}$ but not to both. The only way to split $\mathbf {x}_{j1}$ apart from $\mathbf {x}_{j2}$ is by a threshold $\epsilon < 10$, but this also splits both $\mathbf {x}_{j1}$ and $\mathbf {x}_{j2}$ from $\mathbf {x}_{i}$. SSDBSCAN handles this label inconsistency scenario by not labeling $\mathbf {x}_{i}$ at all, leaving it unclustered as *noise*.

#### Algorithmic approach

Algorithmically, SSDBSCAN does not pre-compute the $\text {MST}_r$. Instead, SSDBSCAN runs an OPTICS search starting from each labeled object and assigning the corresponding label temporarily to the unlabeled objects as they are found and processed by the OPTICS ordering traversal. However, this procedure, called label expansion, uses the mutual reachability distance in Definition 8, rather than the original, asymmetric version of OPTICS in Definition 6. The use of a symmetric distance makes OPTICS algorithmically identical to Prim’s algorithm to compute MSTs. From this perspective, SSDBSCAN dynamically builds an MST in the space of mutual reachability distances, starting from each object $\mathbf {x}_{j1} \in \mathbf {X}_{L}$ and provisionally assigning its label ${{\,\mathrm{class}\,}}(\mathbf {x}_{j1})$ to the traversed unlabeled objects until an object $\mathbf {x}_{j2} \in \mathbf {X}_{L}$ with a different label, ${{\,\mathrm{class}\,}}(\mathbf {x}_{j2}) \ne {{\,\mathrm{class}\,}}(\mathbf {x}_{j1})$, is found. When such an object is found, SSDBSCAN backtracks and only confirms the labels assigned to objects before the largest edge on the path from $\mathbf {x}_{j1}$ to $\mathbf {x}_{j2}$, as these objects are “closer” to $\mathbf {x}_{j1}$ than to $\mathbf {x}_{j2}$ from a density-connectivity perspective, and they can be separated from those objects beyond such a largest edge (including $\mathbf {x}_{j2}$) by any threshold $\epsilon$ smaller than this edge’s weight.

SSDBSCAN stops when the label expansion procedure has been run from each $\mathbf {x}_{j} \in \mathbf {X}_{L}$. Objects not labeled by any of the OPTICS initializations are left unclustered as noise. These objects are those for which no density threshold exists that can make them part of any cluster without incurring label inconsistency.

#### Shortcomings

SSDBSCAN has a number of shortcomings that will be addressed later in this work:

1. **Order-dependency:** If the $\text {MST}_r$ is not unique, i.e., when there are different yet equally optimal (minimum) spanning trees in the transformed space of mutual reachability distances, some objects may end up with different labels depending on the order of the various OPTICS traversals. The reason is that, for different traversals, the implicit minimum spanning trees that are partially and dynamically built from different starting vertices (labeled objects) may be different, and these differences can be shown to possibly cause the algorithm to be order-dependent. Order-dependency may also occur when there is more than one largest edge (i.e., a tie) on the sub-path of the $\text {MST}_r$ between two labeled objects that have different labels;
2. **Re-computations:** rather than pre-computing the $\text {MST}_r$, SSDBSCAN implicitly and partially builds it from different starting vertices. Clearly, many portions of the $\text {MST}_r$ are likely to be recomputed multiple times, which is unnecessarily inefficient from a computational point of view;
3. **Noise and missing clusters:** from a *clustering* perspective, the fact that some objects are left unclustered as noise is expected, especially from a density-based perspective. In SSDBSCAN, however, entire clusters may be left unclustered as noise, typically when they do not contain any labeled object and are well-separated from other clusters, closer to each other, containing objects with different labels, such that the missed clusters cannot be reached at a density level without incurring violations of the label consistency assumption (Assumption 5). This is why SSDBSCAN assumes that there is “at least one (possibly more) labeled object from each class” (Assumption 4), which is, however, a *classification* rather than a clustering assumption. From a classification perspective, even when no cluster is missed, leaving a fraction of objects unlabeled as noise (typically global or local outliers) may be undesired. This particular issue is not present in a related algorithm, HISSCLU, which we discuss next.

### HISSCLU

HISSCLU (Böhm and Plant 2008) can be seen as a semi-supervised version of OPTICS that produces, instead of the original, purely unsupervised reachability plot, a colored version of the plot where every unlabeled object of the dataset is assigned a class label (i.e., a color) from the labeled set, $\mathbf {X}_{L} \subset \mathbf {X}$. The colored plot provides a visual contrast between the clustering structure, revealed as valleys and peaks following an OPTICS ordering ($\prec$), and the transductive classification, mapped as colors in the plot. The main algorithm consists of two stages, a **preprocessing stage** and a **label expansion stage**, as described next.

#### Preprocessing stage (label-based distance weighting)

Before any label expansion takes place, HISSCLU pre-computes a weight for each pairwise distance $d(\mathbf {x}_{i},\mathbf {x}_{j})$ in the dataset. The resulting, weighted distances are used in lieu of the original (e.g., Euclidean) distances in the subsequent steps of the algorithm, namely, reachability distance computations and OPTICS-based label propagation. Such a preprocessing stage is designed to widen the gap between nearby classes by stretching distances between objects around class boundaries, thus allowing the algorithm to expand different class labels even in situations where no natural boundaries of low density between different classes exist. The mathematical and algorithmic details are omitted here for the sake of compactness, but the basic intuition is the following: a pair of objects $(\mathbf {x}_{p},\mathbf {x}_{q}) \in \mathbf {X}_{L}\times \mathbf {X}_{L}$ for which ${{\,\mathrm{class}\,}}(\mathbf {x}_{p}) \ne {{\,\mathrm{class}\,}}(\mathbf {x}_{q})$ establishes a separating hyperplane that perpendicularly crosses the midpoint on the line segment between these two objects (as points in an Euclidean space). A pair of objects $(\mathbf {x}_{i},\mathbf {x}_{j})$ on different sides of this hyperplane in the vicinity of $\mathbf {x}_{p}$ and $\mathbf {x}_{q}$ will have their distance stretched by a multiplicative weight $\ge 1$. The closer to the hyperplane, the greater the weight (stretching). The maximum weight is a user-defined parameter, $\rho \ge 1$ ($\rho = 1$ means no weighting). The weighting decays towards the minimum value 1 with an increasing distance of the objects from the separating hyperplane. Objects “behind” $\mathbf {x}_{p}$ and $\mathbf {x}_{q}$ in relation to the hyperplane are not affected (unitary weight). The shape and rate of decay is controlled by a second parameter, $\xi > 0$ ($\xi = 1$ gives a parabolic decrease, $\xi > 1$ gives a faster, bell-shaped decrease corresponding to a sharper “influence region” around the separating hyperplane, whereas $0< \xi < 1$ gives a more square-shaped decrease, i.e., a wider influence region around the separating hyperplane). This mechanism allows HISSCLU to expand different class labels even within clusters that are formed by more than one class, i.e., clusters of objects that are density connected but not pure in their labels.

#### Label expansion stage

Conceptually, the transductive classification performed by HISSCLU is essentially the label propagation procedure described in Definition 9, which also serves as a basis for SSDBSCAN as previously discussed in Sect. 3.2. However, unlike SSDBSCAN, HISSCLU does not make the label consistency assumption (Assumption 5), thus being able to assign a label to every unlabeled object in $\mathbf {X}_{U} \subset \mathbf {X}$. In other words, no object is left unlabeled as noise.

**Fig. 2:**
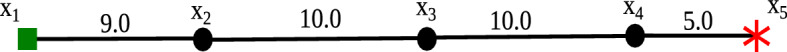
Label assignment in HISSCLU: object $\mathbf {x}_{2}$ is assigned to $\mathbf {x}_{1}$ (green square), whereas $\mathbf {x}_{3}$ and $\mathbf {x}_{4}$ are assigned to $\mathbf {x}_{5}$ (red star) (Color figure online)

But if noise is not an option, how does HISSCLU handle possible label inconsistencies, which can be caused by ties w.r.t. the maximum $\text {MST}_r$ edge (referred to in the definition)? For instance, let us consider the illustrative example in Fig. 2, where we have 5 data objects and the corresponding $\text {MST}_r$. The label propagation procedure described in Definition 9 would arguably assign ${{\,\mathrm{class}\,}}(\mathbf {x}_{2}) = {{\,\mathrm{class}\,}}(\mathbf {x}_{1})$ (green square) and ${{\,\mathrm{class}\,}}(\mathbf {x}_{4}) = {{\,\mathrm{class}\,}}(\mathbf {x}_{5})$ (red star). The middle point ($\mathbf {x}_{3}$), however, is undefined, as there is a tie in the largest edge (10) from this point to objects with different labels. In SSDBSCAN, this point should be left unlabeled (noise), because there is no density threshold that can keep it density-connected to either $\mathbf {x}_{1}$ or $\mathbf {x}_{2}$ but not to both.^2^ Differently, HISSCLU uses instead the 2nd largest edge to resolve the tie (then the 3rd if there is another tie, and so on). In our example, the 2nd largest edge is 9 (green square) versus 5 (red star), thus ${{\,\mathrm{class}\,}}(\mathbf {x}_{3}) = {{\,\mathrm{class}\,}}(\mathbf {x}_{5})$ (red star).

Algorithmically, HISSCLU operates similarly to SSDBSCAN in the sense that it also runs an OPTICS search starting from each labeled object in $\mathbf {X}_{L}$ and assigning labels temporarily to unlabeled objects as they are found by the OPTICS ordering traversal. Unlike SSDBSCAN, however, the traversal does not stop and backtracks once an object with a different label is found. More importantly, the OPTICS traversal in HISSCLU occurs*simultaneously* from all labeled objects. To that end, the OPTICS priority queue is initialized with every unlabeled object having its smallest reachability distance from a labeled object, say $\mathbf {x}_{j} \in \mathbf {X}_{L}$, as its priority key, and ${{\,\mathrm{class}\,}}(\mathbf {x}_{j})$ as its temporary label. This corresponds to initializing OPTICS from all objects in $\mathbf {X}_{L}$ at the same time, rather than from a single object. Each object $\mathbf {x}_{o}$ removed from the queue is processed and its temporary label becomes permanent. The reachability distance from $\mathbf {x}_{o}$ to every object $\mathbf {x}_{i}$ still unprocessed inside the queue is computed, and whenever $\mathbf {x}_{i}$ is reached by a processed object $\mathbf {x}_{o}$ with reachability distance smaller than $\mathbf {x}_{i}$’s current priority key, $\mathbf {x}_{i}$’s key is updated, its temporary label is set to ${{\,\mathrm{class}\,}}(\mathbf {x}_{o})$, and the priority queue is rearranged accordingly. This procedure has been shown (Böhm and Plant 2008) to ensure that the final labels respect the desired “min-max” reachability notion, resolving ties in the maximum edge as described above. However, HISSCLU is still subject to issues previously discussed in the context of SSDBSCAN (Sect. 3.2.3), which relate to the fact that (possibly different) minimum spanning trees are built partially and dynamically, rather than pre-computed.

#### Flat clustering extraction (k-clustering)

Like OPTICS, the reachability plot that results from HISSCLU encodes only visually and *implicitly* a density-based clustering hierarchy. The colors in the plot, in turn, represent a transductive classification of the data from the collection of pre-labeled objects, rather than a clustering result. For scenarios where an explicit clustering solution is desired, Böhm and Plant (2008) offer an optional, post-processing flat clustering extraction stage of HISSCLU, called *k-clustering*, which essentially applies an arbitrary global density threshold to perform a conventional horizontal cut through the reachability plot, analogous to extracting DBSCAN solutions from OPTICS.

### HDBSCAN*

HDBSCAN* (Campello et al. 2013a) is a hierarchical algorithm for unsupervised density-based clustering, which has also been extended to perform hierarchy simplification and visualization, optimal non-hierarchical clustering, and outlier detection (Campello et al. 2015). In the following we describe the core algorithm and extensions that are relevant in our context.

#### Basic algorithm

Following Hartigan’s principles of density-contour clusters and trees (Hartigan 1975), the core HDBSCAN* algorithm provides as a result a complete hierarchy composed of all possible DBSCAN* clustering solutions (as defined in Sect. 3.1) for a given value of $m_{\text {pts}}$ and an infinite range of density thresholds, $\epsilon \in [0,\infty )$, in a nested (i.e., dendrogram-like) way. Key to achieving this is the following transformed proximity graph (*conceptual* only, it does not need to be materialized) (Campello et al. 2013a, 2015):

##### Definition 10

(*Mutual reachability graph*) The *mutual reachability graph* is a complete graph, $G_{m_{\text {pts}}}$, in which the objects of ${\mathbf X}$ are vertices and the weight of each edge is the mutual reachability distance (w.r.t. $m_{\text {pts}}$) between the respective pair of objects.

Let $G_{m_{\text {pts}},\epsilon } \subseteq G_{m_{\text {pts}}}$ be the graph obtained by removing all edges from $G_{m_{\text {pts}}}$ having weights greater than some value of $\epsilon$. From our previous discussions it is clear that clusters according to DBSCAN* w.r.t. $m_{\text {pts}}$ and $\epsilon$ are the connected components of core objects in $G_{m_{\text {pts}},\epsilon }$, whereas the remaining objects are noise. This observation allows to produce all DBSCAN* clusterings for any $\epsilon \in [0,\infty )$ in a nested, *hierarchical* way by removing edges in decreasing order of weight from $G_{m_{\text {pts}}}$.

Notice that this is essentially the graph-based definition of the hierarchical Single-Linkage algorithm (Jain and Dubes 1988), and therefore there is a conceptual relationship between the algorithms DBSCAN* and Single-Linkage in the *transformed space of mutual reachability distances*: the clustering obtained by DBSCAN* w.r.t. $m_{\text {pts}}$ and some value $\epsilon$ is identical to the one obtained by first running Single-Linkage on the transformed space of mutual reachability distances (w.r.t. $m_{\text {pts}}$), then, cutting the resulting dendrogram at level $\epsilon$ of its scale, and treating all resulting singletons with $d_{\text {core}} > \epsilon$ as noise. This suggests that we could implement a hierarchical version of DBSCAN* by applying an algorithm that computes a Single-Linkage hierarchy on the *transformed space* of mutual reachability distances.

One of the fastest ways to compute a Single-Linkage hierarchy is by using a divisive algorithm that works by removing edges from a minimum spanning tree in decreasing order of weights (Jain and Dubes 1988), here, corresponding to mutual reachability distances, i.e., edges from the $\text {MST}_r$. HDBSCAN* augments the ordinary $\text {MST}_r$ with self-loops whose weights correspond to the core distance of the respective object (vertex), to directly represent the level in the hierarchy below which an isolated object is a noise object ($d_{\text {core}}> \epsilon$), and above which it may be part of a cluster or a cluster on its own, i.e., a dense singleton. In short, the core HDBSCAN* is as follows: the $\text {MST}_r$ is computed using some computationally efficient method (e.g. Prim’s), augmented with self-edges, then edges are removed in decreasing order and the resulting connected components are labeled as clusters. In case of ties, edges are removed simultaneously.

Notice that, unlike SSDBSCAN, which also makes use of the $\text {MST}_r$, HDBSCAN* provides a hierarchical rather than a flat clustering solution, and unlike OPTICS and HISSCLU, whose reachability plots only *implicitly* encode DBSCAN clustering solutions for a given value of $m_{\text {pts}}$ and $\epsilon \in [0,\infty )$, the corresponding hierarchical relations in HDBSCAN* are explicit and readily available.

#### HDBSCAN* with all-points core distance

 Campello et al. (2013a, 2015) showed that the parameter $m_{\text {pts}}$, which is commonly shared by all density-based algorithms previously discussed, corresponds to a classic smoothing factor of a nonparametric, nearest neighbors density estimate. This parameter is not critical and can be useful to provide the user with fine-tuning control of the results, e.g., by visual inspection of the clustering hierarchies or of the reachability plots. It can be removed though, if desired, basically by replacing the core and mutual reachability distances in Definitions 5 and 8 with a parameterless alternative. In particular, a parameterless version of HDBSCAN* was proposed (Moulavi 2014) that is based on a new core distance of an object, which does not depend on its $m_{\text {pts}}$-neighborhood, but rather considers the dataset in a way that closer objects contribute more to the density than farther objects do:

##### Definition 11

(*All-points core distance*) The all-points core-distance of a *d*-dimensional point $\mathbf {x}$ of a dataset $\mathbf {X}$ with respect to all other $n-1$ points in $\mathbf {X}$, i.e., $\mathbf {X} \backslash \{\mathbf {x}\}$, is defined as (Moulavi 2014):

$$\begin{aligned} d_{\text {aptsCore}}(\mathbf {x}) = \left( \frac{ \sum _{ \mathbf {x}_{i} \in \mathbf {X} \backslash \{ \mathbf {x}\} } \left( \frac{1}{ d(\mathbf {x},\mathbf {x}_{i}) } \right) ^{d} }{n-1} \right) ^{-\frac{1}{d} } \end{aligned}$$

Let us note that the all-points core distance is only meaningful for datasets as points in a *d*-dimensional real vector space, as opposed to the original HDBSCAN* (as well as DBSCAN, OPTICS, and SSDBSCAN) that can operate with any dataset for which some type of pairwise distance between objects can be defined. HISSCLU shares the same limitation when its preprocessing stage is required.

A summary table with the properties and assumptions of all the density-based algorithms studied in this paper, including our new algorithms for semi-supervised classification and clustering (to be introduced in Sects. 4 and 5, respectively), is provided in “Appendix”.

## Unified framework for density-based classification

In the previous section, we elaborated how previous clustering and semi-supervised clustering algorithms in the density-based clustering paradigm can all conceptually be viewed as processing minimum spanning trees in a space of reachability distances, i.e., processing MSTs of a conceptual, complete graph where the nodes are the objects, and the edge weights are the reachability distances between objects. These reachability distances can be based on unmodified or modified (e.g., weighted, streched) distances between objects.

In this section, we present a new framework for semi-supervised classification by extending the HDBSCAN* clustering framework with additional, optional steps, derived from “decoupled” building blocks of the algorithms discussed in Sect. 3, so that these building blocks can be re-combined and applied in different ways. This will allow us to study the performance gain of each building block and specify different instances for semi-supervised classification—one can be considered a close approximation of HISSCLU, and another one is a looser, but faster approximation, others are novel variants that have not been investigated before.

### The components of the framework (building blocks)

The building blocks for semi-supervised classification in our framework are the following:

1. **The adopted definition of core and reachability distances:** In our framework, we will study both the standard definition of core-distance with the parameter $m_{\text {pts}}$, which has been adopted by all the algorithms discussed in Sect. 3 (Definition 5), as well as the parameter-free all-points core distance (Definition 11). Given a notion of core distance, we will only use the symmetric notion of *mutual* reachability distance (Definition 8) as used by SSDBSCAN, DBSCAN*, and HDBSCAN*, even though the “older” algorithms HISSCLU, DBSCAN, and OPTICS are based on the asymmetric notion in Definition 6. The reason is that the mutual reachability distance has a statistically more sound interpretation, is simpler and, in practice, the difference in results tends not to be very noticeable.
2. **MST computation in the space of mutual reachability distances:** Recall from Sect. 3 that SSDBSCAN and HISSCLU compute multiple MSTs “on-the-fly”, starting from labeled objects. Such an approach is inefficient and, from a conceptual point of view, not necessary. It is obviously not necessary when the MST of the conceptual, complete graph in the transformed space of mutual reachability distances is unique; then the same MST will just be re-computed multiple times. But even when the MST is not unique, the different MSTs are in a sense equivalent from the perspective of representing the inherent cluster structure of a data set: none of them should lead to fundamentally different conclusions about the density distribution of the dataset. Therefore, in our framework we explicitly decouple the MST construction from the label expansion (as we have already done conceptually in the discussion of the algorithms SSDBCAN and HISSCLU in the previous section), and we will use HDSBCAN*’s efficient algorithm to compute the “extended” $\text {MST}_r$ as described in Sect. 3.4.
3. **Label expansion:** Given a computed graph $\text {MST}_r$, in our framework for semi-supervised classification we implement a label expansion method on top of the $\text {MST}_r$, similar to HISSCLU (see Sect. 3.3.2), but using the single $\text {MST}_r$ computed by HDBSCAN* to propagate the labels based on the path with the smallest largest edge to a labeled object, resolving ties, possibly consecutively, by considering the smaller of the next largest edge on the paths. This can be implemented by starting with a “connected component” $C_i$ for each pre-labeled object $\mathbf {x}_i \in \mathbf {X}_L$ that initially contains only $\mathbf {x}_i$. These connected components $C_i$ are then iteratively extended by traversing the $\text {MST}_r$ in a way that ensures correctness of the final result (Gertrudes et al. 2018). A detailed description of the algorithm alongside with a pseudo-code is provided in “Appendix”.

**Fig. 3:**
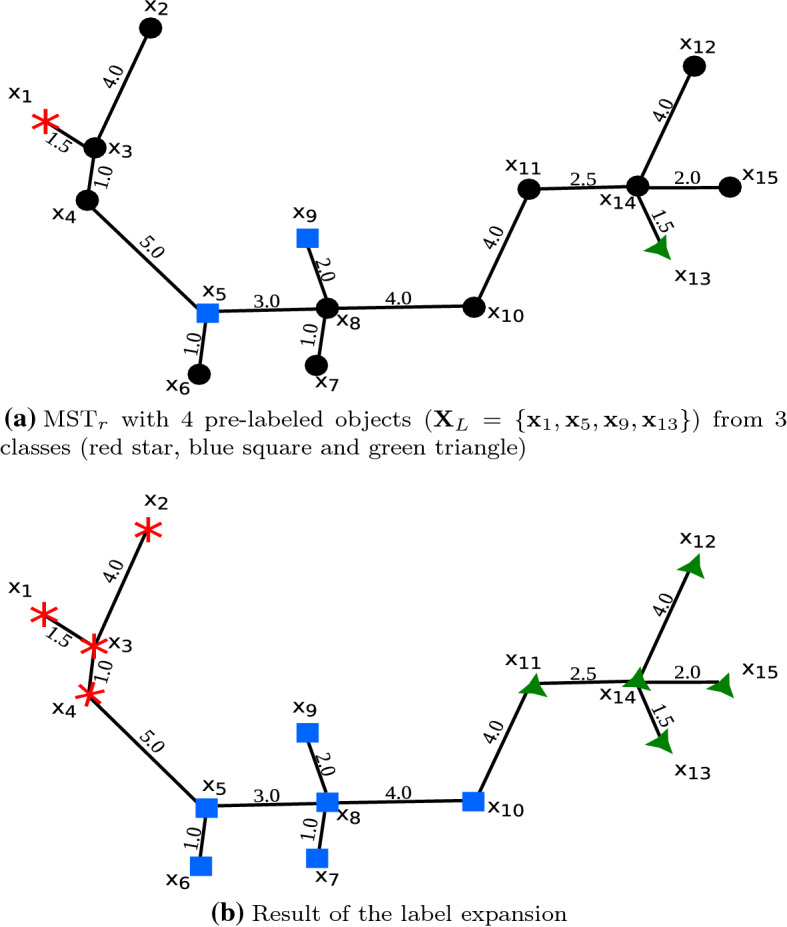
Example of density-based label expansion (Color figure online)

*Example:* Figure 3 illustrates our label expansion with an example. Figure 3a displays 15 objects and their minimum spanning tree in the mutual reachability distance space ($\text {MST}_r$). To perform the label expansion, we initialize connected components with the pre-labeled objects, $C_1 = \{\mathbf {x}_{1}\}$, $C_2 = \{\mathbf {x}_{5}\}$, $C_3 = \{\mathbf {x}_{9}\}$, and $C_4 = \{\mathbf {x}_{13}\}$.

- From all the components, the first edge to be analyzed is the one connecting $\mathbf {x}_{5}$ to $\mathbf {x}_{6}$ since it is the one with the lowest weight, 1.0, among all of the currently “outgoing” edges of the current components. Since $\mathbf {x}_{6}$ is not labeled yet, it will receive the label of $C_{2}$ (i.e., ${{\,\mathrm{class}\,}}(\mathbf {x}_{5})$), $\mathbf {x}_{6}$ is added to $C_{2}$, and since there is no other edge incident to $\mathbf {x}_{6}$, no new outgoing edges are added.
- Then, the edges with the next largest edge weight, 1.5, connecting to $C_1, \ldots , C_4$, which are the edges connecting $\mathbf {x}_{1}$ to $\mathbf {x}_{3}$ and connecting $\mathbf {x}_{13}$ to $\mathbf {x}_{14}$, are selected. The two edges connect to a different, not-yet-labeled object and thus $\mathbf {x}_{3}$ is labeled with ${{\,\mathrm{class}\,}}(\mathbf {x}_{1})$ and added to $C_1$, while $\mathbf {x}_{14}$ is labeled with ${{\,\mathrm{class}\,}}(\mathbf {x}_{13})$ and added to $C_4$.
- Two new unprocessed edges are now incident to $C_1$, connecting $\mathbf {x}_{2}$ and $\mathbf {x}_{4}$ to $\mathbf {x}_{3}$, with edge weights of 4.0 and 1.0, respectively; three new unprocessed edges are now incident to $C_4$, connecting $\mathbf {x}_{11}$, $\mathbf {x}_{12}$, and $\mathbf {x}_{15}$ to $\mathbf {x}_{14}$, with edge weights of 2.5, 4.0, and 2.0, respectively. The smallest edge weight of all the outgoing edges of the current connected components is now 1.0 on the edge connecting $\mathbf {x}_{4}$ to $\mathbf {x}_{3}$. Since $\mathbf {x}_{4}$ is unlabeled, it is labeled with ${{\,\mathrm{class}\,}}(\mathbf {x}_{1})$ and added to $C_1$, and the edge connecting $\mathbf {x}_{4}$ to $\mathbf {x}_{5}$ is added to the outgoing edges of $C_1$.
- The now smallest edge weight of outgoing edges is 2.0 on the edges connecting $\mathbf {x}_{9}$ to $\mathbf {x}_{8}$ and connecting $\mathbf {x}_{14}$ to $\mathbf {x}_{15}$. The two edges are incident to two different unlabeled objects. Hence $\mathbf {x}_{8}$ is added to $C_3$ (which currently had only $\mathbf {x}_{9}$ in it) and receives its label, and $\mathbf {x}_{15}$ is added to $C_4$ (currently with $\mathbf {x}_{13}$ and $\mathbf {x}_{14}$) and receives its label; no new outgoing edges are incident to $C_4$ but three new outgoing edges are incident to $C_3$: ($\mathbf {x}_{8}$, $\mathbf {x}_{7}$), ($\mathbf {x}_{8}$, $\mathbf {x}_{5}$), and ($\mathbf {x}_{8}$, $\mathbf {x}_{10}$), with corresponding edge weights 1.0, 3.0, and 4.0.
- Next, the smallest edge weight of outgoing edges is now 1.0 on the edge ($\mathbf {x}_{8}$, $\mathbf {x}_{7}$); $\mathbf {x}_{7}$ is added to $C_3$ and receives its label, no new outgoing edges are incident to $\mathbf {x}_{7}$. Similarly, $\mathbf {x}_{11}$ is added to $C_4$ since its edge weight of 2.5 is the next smallest.
- After adding $\mathbf {x}_{11}$ to $C_4$, $C_4$ has a new outgoing edge, connecting $\mathbf {x}_{11}$ to $\mathbf {x}_{10}$, with edge weight 4.0. Next, the smallest edge weight of outgoing edges is now 3.0 on the edge between $\mathbf {x}_{5}$ and $\mathbf {x}_{8}$, outgoing from $C_2$ as well as $C_3$. This edge connects two components with the same label. Hence $C_2$ and $C_3$ are merged into and replaced by $C_{2\_3}$.
- In the next step the smallest edge weight of outgoing edges is 4.0 on the edges ($\mathbf {x}_{3}$, $\mathbf {x}_{2}$), outgoing from $C_1$, ($\mathbf {x}_{8}$, $\mathbf {x}_{10}$), outgoing from $C_{2\_3}$, ($\mathbf {x}_{11}$, $\mathbf {x}_{10}$) outgoing from $C_4$, and ($\mathbf {x}_{14}$, $\mathbf {x}_{12}$), outgoing from $C_4$. Similar to previous cases in which an unlabeled object is connected to just one of the current components, $\mathbf {x}_{2}$ is labeled and added to $C_1$, and $\mathbf {x}_{12}$ is labeled and added to $C_4$.
- The unlabeled object $\mathbf {x}_{10}$ is connected to two components with different class labels. The smallest largest edge weight on paths to pre-labeled objects in both $C_{2\_3}$ and $C_{4}$ is 4.0, so the next (2nd) largest edge on all paths to pre-labeled objects is considered, which are 2.0 on the path from $\mathbf {x}_{9}$ in $C_{2\_3}$ to $\mathbf {x}_{10}$, 3.0 on the path from $\mathbf {x}_{5}$ in $C_{2\_3}$ to $\mathbf {x}_{10}$, and 2.5 on the path from $\mathbf {x}_{13}$ in $C_{4}$ to $\mathbf {x}_{10}$. The smallest of these is 2.0 from $\mathbf {x}_{9}$, hence $\mathbf {x}_{10}$ is added to $C_{2\_3}$ and obtains its label.
- In the last step, the edge ($\mathbf {x}_{4}$, $\mathbf {x}_{5}$) with edge weight 5.0 is ignored since it connects two components with different class labels.

Figure 3b shows the final result.

4. **Preprocessing: Label-based distance weighting:** As an optional step, one may want to compute weighted distances based on the labeled subset of the data, as described in Sect. 3.3.1 for HISSCLU. We include such a step in our framework that applies label-based distance weighting on all the pairwise distances before computing core and reachability distances, as in HISSCLU. However, this step can be computationally time consuming. As an approximation, we also propose to apply distance weighting *after* the $\text {MST}_r$ has been constructed, so that it only needs to be applied to the mutual reachability distances of the edges in the $\text {MST}_r$.

### The framework

The above building blocks can be combined in different ways to obtain different and novel semi-supervised classification methods as instances of our framework, through which we can study the contribution of each building block in an overall approach to semi-supervised classification. We denote different instances using the notation HDBSCAN*(core-distance-definition, label-based-distance-weighting-scheme), where *core-distance-definition* stands either for the standard core distance definition, abbreviated by “cd”, or the all-points core distance, abbreviated by “ap”; and *label-based-distance-weighting-scheme* stands for label-based distance weighting of all pairwise distances, abbreviated as “wPWD”, or label-based distance weighting of the $\text {MST}_r$ edges, abbreviated as “wMST”; no weighting is denoted as “—”.

**Fig. 4:**
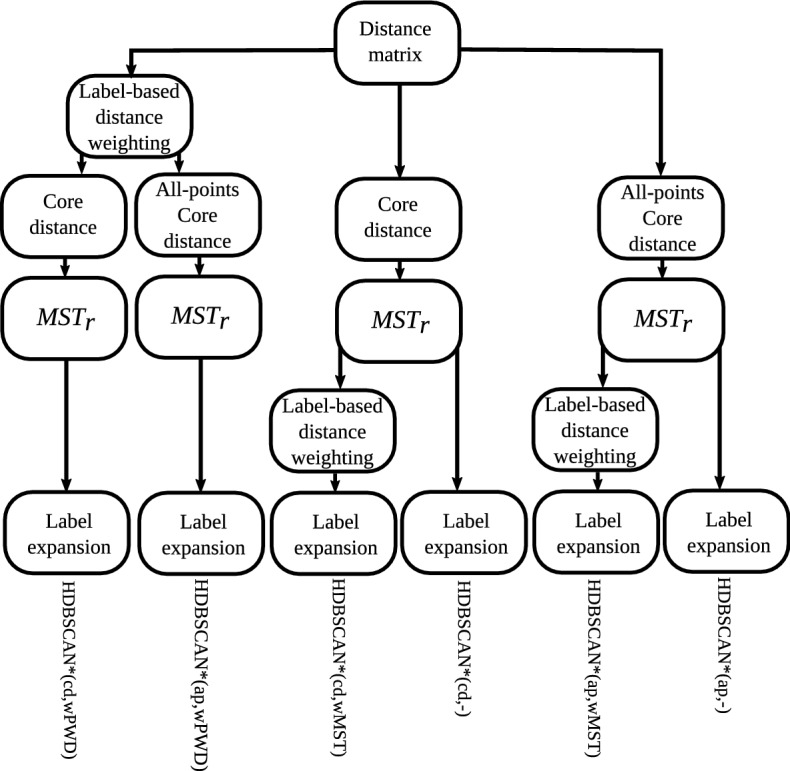
Unified framework of density-based algorithms for semi-supervised classification

Figure 4 presents a schematic view of the instances of our framework for semi-supervised classification, which we will study in the experimental evaluation. Each branch in the diagram starts with a distance matrix as input and represents a different algorithm. In the leftmost branch, label-based distance weighting on the pairwise distances of the distance matrix is performed, standard core-distance is used, $\text {MST}_r$ is computed, and label expansion is performed. This algorithm is denoted by HDBSCAN*(cd,wPWD), and it is similar to HISSCLU, but it uses the symmetric, mutual reachability distance rather than the old, asymmetric version, and relies only on a single pre-computed $\text {MST}_r$. The third branch that uses standard core distance and performs label-based distance weighting only on the $\text {MST}_r$ edges can be considered an even looser but much faster approximation of HISSCLU. The other branches show other methods, using alternatively the all-points core distance, and perform or omit completely the two options for label-based distance weighting.

### Complexity

The asymptotic complexity of instances of our framework is as follows. Given the dataset $\mathbf {X}$, computing the *core* or*all-points-core* distances and the construction of the $\text {MST}_r$ has an overall time complexity of $O(n^{2})$ (this part is the same as HDBSCAN*).

The label expansion takes, in the worst case, $O(n \log n)$ time if the set of outgoing edges of the connected components is maintained in a heap, with edge weights as priority key.

- In the algorithms that apply the label-based weighting function to the entire distance matrix—HDBSCAN*(cd,wPWD) and HDBSCAN*(ap,wPWD)—the additional runtime is of the order $O(n^{2} + |\mathbf {X}_L|n)$, where $|\mathbf {X}_L|$ is the number of pre-labeled objects: the first term is the number of distances that have to be weighted, whereas the second term corresponds to the pre-computation of the elements required to compute any weight in constant time (following the optimized approach of Böhm and Plant (2008)).
- For the algorithms that apply the weighting function in the $\text {MST}_r$ instead—HDBSCAN*(cd,wMST) and HDBSCAN*(ap,wMST)—the additional runtime is $O(n + |\mathbf {X}_L|n) \rightarrow O(|\mathbf {X}_L|n)$ since the $\text {MST}_r$ has only $n-1$ edges to be weighted. Assuming $|\mathbf {X}_L| \ll n$ as usual in semi-supervised classification, the additional runtime of this approach is *O*(*n*), in contrast to $O(n^{2})$ of the original HISSCLU weighting. The former becomes even more attractive when it is necessary to compute the label expansion with different sets of labeled objects. In this case, it will be necessary only to adapt the edges of the $\text {MST}_r$, instead of repeating the process of computing the core distance (or the all-points core distance), and to compute the $\text {MST}_r$ in every different label configuration.

In total, the overall runtime complexity of the algorithms in the framework for semi-supervised classification is hence $O(n^{2})$. If pairwise distances are computed on demand, it requires*O*(*n*) memory only.

## Density-based semi-supervised clustering

When performing semi-supervised *classification*, all classes are known in advance, pre-labeled objects from all of these classes are available, and all unlabeled objects are in principle supposed to be labeled by the algorithm. In contrast, when the task at hand is semi-supervised *clustering*, not all categories are necessarily known in advance, which means that labels may not be available for some (unknown) classes yet to be discovered, and part of the unlabeled objects may be left unclustered as noise. In this case, the framework proposed in Sect. 4 is no longer suitable.

**Fig. 5:**
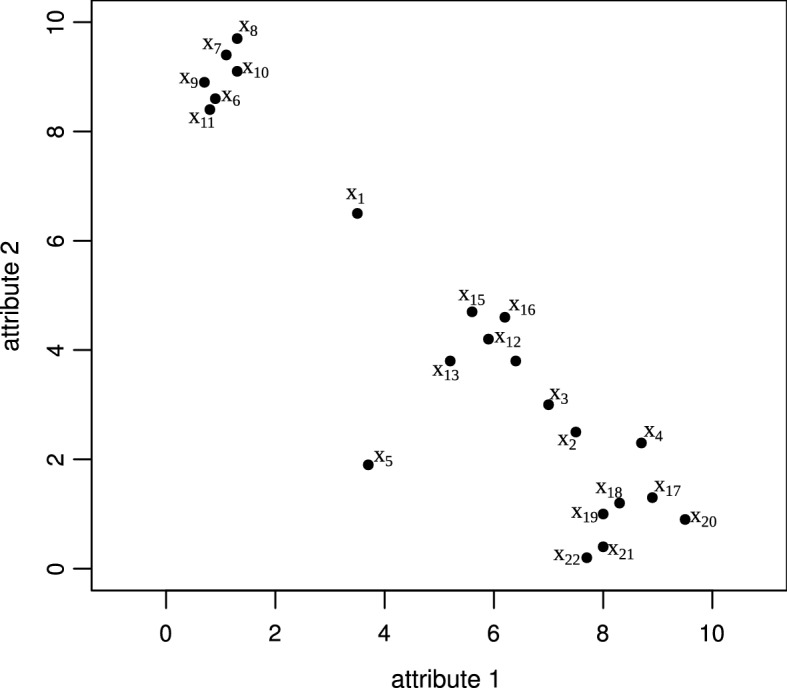
Illustrative dataset

HDBSCAN* offers an optional post-processing method of its clustering hierarchy, called FOSC, that can extract a flat clustering solution by performing local cuts through the hierarchy in order to select a collection of non-overlapping clusters (and, possibly, objects unclustered as noise) that is optimal according to a given unsupervised or semi-supervised criterion. FOSC is unique in that it can perform non-horizontal cuts through a hierarchy, which means that clusters can be extracted from different hierarchical levels. In HDBSCAN*, this means that solutions composed of clusters at various density levels can be obtained, which could not be obtained by a conventional, global horizontal cut at a single hierarchical level. In the following we revisit FOSC as this method plays a central role in our approach for density-based semi-supervised clustering.

### FOSC

FOSC (*Framework for Optimal Extraction of Clusters*) was proposed by Campello et al. (2013b) as a general framework to perform optimal extraction of flat clustering solutions from clustering hierarchies. In order to understand how the method operates in HDBSCAN*, let us consider an example.

Figure 5 shows a toy dataset with 22 objects. The complete clustering hierarchy produced by HDBSCAN* with $m_{\text {pts}} = 3$ is shown in Table 1,^3^ where rows correspond to hierarchical levels (density thresholds for varied $\epsilon$), columns correspond to data objects, and entries contain cluster labels (“0” stands for noise). Notice that, unlike traditional dendrograms, clusters can shrink and yet retain the same label when individual objects (or spurious components with fewer than an optional, user-defined minimum cluster size, $m_{\text {ClSize}}$) are disconnected from them becoming noise, as the density threshold increases for decreasing values of $\epsilon$ (top-down the hierarchy). Only when a cluster is divided into two non-spurious subsets of density-connected objects the resulting subsets are deemed new clusters. This way, the complete hierarchy in Table 1 can actually be represented as a simplified cluster tree where the root ($\mathbf {C}_1$) is the “cluster” containing the whole dataset, which subdivides into two child nodes corresponding to clusters $\mathbf {C}_2$ and $\mathbf {C}_3$, and these further subdivide into two sub-clusters each ($\mathbf {C}_4$ and $\mathbf {C}_5$ from $\mathbf {C}_2$, $\mathbf {C}_6$ and $\mathbf {C}_7$ from $\mathbf {C}_3$).

**Table 1:** Cluster hierarchy with $m_{\text {pts}}=m_{\text {ClSize}}=3$. Colors (red, blue, green, and magenta) highlight a collection of pre-labeled objects, $\mathbf {X}_{L} = \{\mathbf {x}_{1}, \mathbf {x}_{6}, \mathbf {x}_{8}, \mathbf {x}_{15}, \mathbf {x}_{18} \}$, which can be used to extract clusters from the hierarchy in a semi-supervised way (cf. Figure 6) (Color table online)

$\epsilon$		$\mathbf {x}_{5}$	$\mathbf {x}_{20}$	$\mathbf {x}_{2}$	$\mathbf {x}_{3}$	$\mathbf {x}_{13}$	$\mathbf {x}_{14}$	$\mathbf {x}_{12}$		$\mathbf {x}_{16}$	$\mathbf {x}_{4}$	$\mathbf {x}_{22}$	$\mathbf {x}_{17}$		$\mathbf {x}_{19}$	$\mathbf {x}_{21}$		$\mathbf {x}_{9}$	$\mathbf {x}_{11}$	$\mathbf {x}_{7}$		$\mathbf {x}_{10}$
3.30	1	1	1	1	1	1	1	1	1	1	1	1	1	1	1	1	1	1	1	1	1	1
3.19	2	2	2	2	2	2	2	2	2	2	2	2	2	2	2	2	3	3	3	3	3	3
3.18	0	2	2	2	2	2	2	2	2	2	2	2	2	2	2	2	3	3	3	3	3	3
1.24	0	0	2	2	2	2	2	2	2	2	2	2	2	2	2	2	3	3	3	3	3	3
1.22	0	0	0	2	2	2	2	2	2	2	2	2	2	2	2	2	3	3	3	3	3	3
1.17	0	0	0	0	4	4	4	4	4	4	5	5	5	5	5	5	3	3	3	3	3	3
1.00	0	0	0	0	4	4	4	4	4	4	0	5	5	5	5	5	3	3	3	3	3	3
0.98	0	0	0	0	0	4	4	4	4	4	0	5	5	5	5	5	3	3	3	3	3	3
0.85	0	0	0	0	0	0	4	4	4	4	0	5	5	5	5	5	3	3	3	3	3	3
0.82	0	0	0	0	0	0	4	4	4	4	0	0	5	5	5	5	3	3	3	3	3	3
0.72	0	0	0	0	0	0	0	4	4	4	0	0	5	5	5	5	3	3	3	3	3	3
0.63	0	0	0	0	0	0	0	4	4	4	0	0	0	5	5	5	3	3	3	3	3	3
0.61	0	0	0	0	0	0	0	4	4	4	0	0	0	5	5	5	6	6	6	7	7	7
0.60	0	0	0	0	0	0	0	0	0	0	0	0	0	0	0	0	6	6	6	7	7	7
0.51	0	0	0	0	0	0	0	0	0	0	0	0	0	0	0	0	6	6	6	0	0	0
0.00	0	0	0	0	0	0	0	0	0	0	0	0	0	0	0	0	0	0	0	0	0	0

Notice in Table 1 that objects belonging to a parent cluster do not necessarily belong to any of its children, as they may become noise before a cluster splits. For instance, objects $\mathbf {x}_1$, $\mathbf {x}_2$, $\mathbf {x}_5$, and $\mathbf {x}_{20}$ belong to $\mathbf {C}_2$ but not to $\mathbf {C}_4$ or $\mathbf {C}_5$. Technically, each of these objects is assigned an individual node on its own in the cluster tree, in this example all as descendants from $\mathbf {C}_2$.^4^ As we will see later, these singleton nodes can only affect cluster extraction in the semi-supervised scenario, and only if the corresponding objects are pre-labeled.

**Fig. 6:**
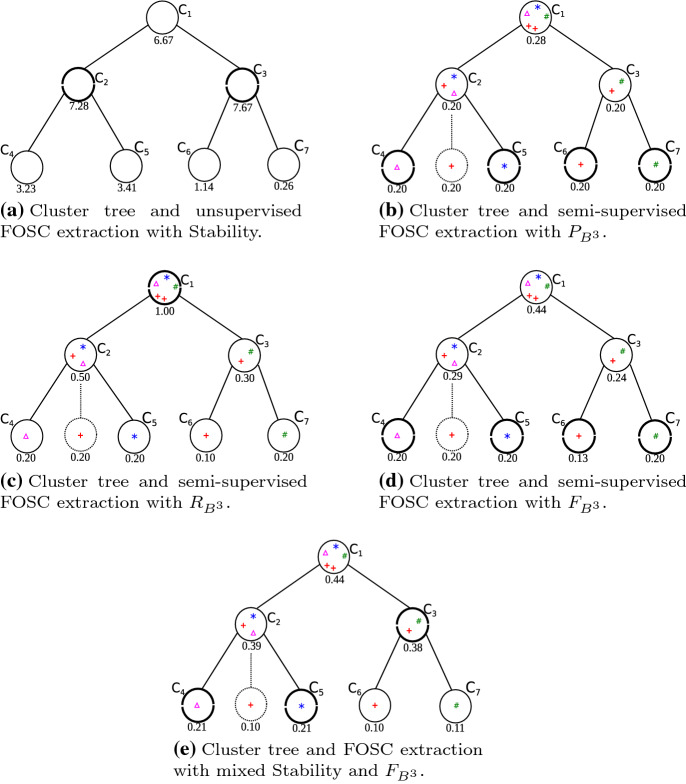
HDBSCAN* cluster tree for the hierarchy in Table 1 and flat clustering extraction using FOSC: **a** unsupervised extraction using Stability;**b**–**d** Labeled-based semi-supervised extraction using $B^3$ Precision, Recall, and F-Measure, respectively; **e** mixed case. The set of pre-labeled objects, $\mathbf {X}_L$, is represented using colored symbols: $\mathbf {x}_1$ and $\mathbf {x}_6$ as red crosses, $\mathbf {x}_8$ as a green hash, $\mathbf {x}_{15}$ as a magenta triangle, and $\mathbf {x}_{18}$ as a blue star. Singleton nodes corresponding to noise objects that are not pre-labeled are omitted as they do not affect computations. Extracted clusters in each case are highlighted in bold (Color figure online)

Figure 6 illustrates the cluster tree corresponding to the clustering hierarchy in Table 1, along with all the information needed to run FOSC in various different unsupervised and semi-supervised settings. Singleton nodes corresponding to noise objects that are not pre-labeled are omitted for the sake of clarity, as they do not affect computations. Notice that the only singleton node displayed, as a dotted circle in Fig. 6b–e, corresponds to object $\mathbf {x}_1$, which is assumed to be pre-labeled (represented as a red cross). Thus it affects computations in the semi-supervised scenarios.

If clusters in the cluster tree can be properly assessed according to a suitable unsupervised or semi-supervised measure of cluster quality, an optimal flat solution in which objects are guaranteed not to belong to more than one cluster can be extracted by FOSC. Formally, let $\{\mathbf {C}_{1}, \ldots , \mathbf {C}_{k}\}$ be the set of all candidate clusters in the cluster tree from which we want to extract a flat solution, $\mathbf {P}$. Assume that there is an objective function $J_T(\mathbf {P})$ that we want to maximize, such that $J_T$ can quantitatively assess the quality of every valid candidate solution $\mathbf {P}$. Functional $J_T(\mathbf {P})$ must be decomposable according to two properties:^5^

1. **Additivity:**$J_T(\mathbf {P})$ must be written as the sum of individual components $J(\mathbf {C}_i)$, each of which is associated with a single cluster $\mathbf {C}_i$ of $\mathbf {P}$;
2. **Locality:** Every component $J(\mathbf {C}_i)$ must be computable locally to $\mathbf {C}_i$, regardless of what the other clusters that compose the candidate solution $\mathbf {P}$ are.

Due to the property of *locality*, the value $J(\mathbf {C}_i)$ associated with every cluster in the cluster tree can be computed beforehand, i.e., prior to the decision on which clusters will compose the final solution to be extracted. These are the values illustrated below each node in Fig. 6.

Due to the property of *additivity*, the objective function can be written as $J_T(\mathbf {P}) = \sum _{\mathbf {C}_i \in \mathbf {P}} J(\mathbf {C}_i)$, and the problem we want to solve is to choose a collection $\mathbf {P}$ of clusters such that: (a) $J_T(\mathbf {P})$ is maximized; and (b) $\mathbf {P}$ is a valid flat solution, i.e., clusters and their sub-clusters are mutually exclusive (no data object belongs to more than one cluster). Mathematically, the optimization problem can be formulated as (Campello et al. 2013b):

1 $$\begin{aligned} \begin{aligned}&\underset{\delta _1, \cdots , \delta _k}{\text {max}}&\sum _{i = 1}^{k} \delta _{i}J(\mathbf {C}_{i}) \\&\text {s.~t.}&\delta _{i} \in \{0,1\}, \; i = 1, \ldots , k. \\&\sum _{j \in \mathbf {I}_{h}} \delta _{j} = 1, \; \forall h~\text {such that }\mathbf {C}_{h}\text { is a leaf node/cluster.} \end{aligned} \end{aligned}$$

where $\delta _{i}~(i = 1, \cdots , k)$ is an indicator function that denotes whether cluster $\mathbf {C}_i$ is selected to be part of the solution $(\delta _{i} = 1)$ or not $(\delta _{i} = 0)$, and $I_h$ is the set of cluster indices on the path from any external node $\mathbf {C}_h$ up to the root $\mathbf {C}_1$. Note that the constraints ensure that a single cluster is selected in any branch from the root to a leaf.

FOSC solves this problem taking advantage of the fact that, due to the locality property of the cluster quality measure *J*, the partial selections made inside any subtree remain optimal in the context of larger trees containing that subtree. This allows for a very efficient, globally optimal dynamic programming method that traverses the cluster tree bottom-up starting from the leaves, comparing the quality of parent clusters against the aggregated quality of the respective subtrees, carrying the optimal choices upwards until the root is reached.

In Fig. 6a, notice that the sum of $J(\mathbf {C}_4) = 3.23$ and $J(\mathbf {C}_5)= 3.41$ (as well as the hidden singleton nodes descending from $\mathbf {C}_2$, all of which have *J* value of zero) is equal to 6.64, which is smaller than $J(\mathbf {C}_2) = 7.28$, hence $\mathbf {C}_2$ is temporarily selected while its subtrees are discarded. Analogously, the aggregated value of $J(\mathbf {C}_6)$ and $J(\mathbf {C}_7)$ (1.4) is compared against $J(\mathbf {C}_3) = 7.67$, which is larger, hence $\mathbf {C}_3$ is temporarily selected whereas $\mathbf {C}_6$ and $\mathbf {C}_7$ are discarded. Now, the sum of $J(\mathbf {C}_2)$ and $J(\mathbf {C}_3)$ (14.95) is larger than $J(\mathbf {C}_1) = 6.67$, hence $\mathbf {C}_1$ is discarded while $\mathbf {C}_2$ and $\mathbf {C}_3$ are retained. Since the root has been reached, the final solution is $\mathbf {P} = \{\mathbf {C}_2, \mathbf {C}_3\}$, with $J_T(\mathbf {P}) = 14.95$.

While many clustering quality criteria from the literature satisfy the additive property, it is not easy to find criteria that satisfy the locality property required by FOSC. In the original publication (Campello et al. 2013b), a criterion was introduced that is based on the classic notion of *cluster lifetime*. The lifetime of a cluster in a clustering dendrogram is basically the length of the dendrogram scale along which the cluster exists (Jain and Dubes 1988). More prominent clusters persist longer across multiple hierarchical levels, so they have a longer lifetime.

This concept has been adapted by Campello et al. (2013b) to account for the fact that in certain hierarchies, including density-based hierarchies such as the one in Table 1, not all data objects stay in the cluster during its whole lifetime, because some objects become noise along the way. In other words, objects have different lifetimes as part of a cluster. The unsupervised measure of *Stability* of a cluster as proposed by Campello et al. (2013b) is the sum of the lifetimes of every object in that cluster, $J(\mathbf {C}_i) = \sum _{\mathbf {x}_j \in \mathbf {C}_i} {{\,\mathrm{lifetime}\,}}(\mathbf {x}_j)$.

For example, in Table 1, cluster $\mathbf {C}_{4}$ appears bottom-up at level 0.61 (formed by objects $\mathbf {x}_{12}$, $\mathbf {x}_{15}$, and $\mathbf {x}_{16}$) and disappears when it gets merged with cluster $\mathbf {C}_{5}$, giving rise to $\mathbf {C}_{2}$ at level 1.22. Along this interval, another three objects join this cluster, at levels 0.82 ($\mathbf {x}_{14}$), 0.98 ($\mathbf {x}_{13}$), and 1.00 ($\mathbf {x}_{3}$). Hence, its Stability is given by $J(\mathbf {C}_{4}) = 3*(1.22 - 0.61) + (1.22 - 0.82) + (1.22 - 0.98) + (1.22 - 1.00) = 2.69$.

To perform flat cluster extraction in an *unsupervised* way, HDBSCAN* uses FOSC with the Stability criterion as described above, except that it replaces $\epsilon$ with $\frac{1}{\epsilon }$ in the scale (which is therefore flipped) for the computation of lifetime. This makes Stability more statistically sound in the density-based context as it becomes equivalent to the concept of relative *excess of mass* of a cluster (Campello et al. 2015).^6^ In this case, the Stability of cluster $\mathbf {C}_{4}$ in our example above would be computed as $J(\mathbf {C}_{4}) = 3*(1/0.61 - 1/1.22) + (1/0.82 - 1/1.22) + (1/0.98 - 1/1.22) + (1/1.00 - 1/1.22) = 3.23$. These are precisely the values show below each cluster in Fig. 6a.

The Stability of any singleton node containing a noise object is defined as zero as the lifetime of noise is undefined. This way, singleton nodes with noise do not directly affect unsupervised cluster extraction. Indirectly though, larger amounts of noise in a final solution $\mathbf {P}$ are indirectly penalized because noise objects do not add anything to the Overall Stability of that solution.

FOSC can operate in a *semi-supervised* way if a suitable measure that takes into account semi-supervision is provided. However, the existing method, currently used by HDBSCAN*, is based on the maximization of the number of (soft, as preferences only) should-link and should-not-link *constraints* that are satisfied in the extracted clusters (Campello et al. 2013b, 2015). Obviously, given a collection of labeled objects, one can produce constraints by creating should-link relations between pairs of objects with the same label, and should-not-link relations between pairs of objects with different labels. Nevertheless, there are two main disadvantages in working with pairwise constraints, rather than directly with labels. The first one is the additional effort to generate the constraints, which is actually unnecessary as we will discuss later (Sect. 5.2). Second, as the number of different labels increase, so does the imbalance between the number of should-not-link constraints and the number of should-link constraints that follow from the labels, as the number of should-not-link constraints increases much stronger with adding additional labels than the number of should-link constraints. Thus the maximization of the number of constraints satisfied tends to be biased towards satisfying should-not-link relations, which may produce unexpected results. As a matter of fact, it has been observed in the semi-supervised clustering literature that adding constraints may possibly decrease the performance of clustering algorithms (Davidson et al. 2006).

### Label-based semi-Supervised FOSC

Here, we introduce a new semi-supervised quality measure for the optimal cluster extraction procedure (FOSC) used by HDBSCAN* that operates directly with labels rather than constraints. We also describe how this measure can be combined with the unsupervised measure of cluster *Stability* (see Sect. 5.1) for optimal cluster extraction, making the resulting combination effective irrespective of whether only part, all or none of the clusters in the data are represented by labeled observations.

Our newly proposed semi-supervised measure of cluster quality is based on the $B^{3}$*Precision* and $B^{3}$*Recall* criteria originally proposed by Bagga and Baldwin (1998) and subsequently studied by Amigó et al. (2009) in the context of an external cluster validation index, called $\text {B}^{3}$ (BCubed). These criteria take pairs of objects into account, but they are computed individually for each pre-labeled object $\mathbf {x} \in \mathbf {X}_L$. Specifically, the $\text {B}^{3}$ Precision of $\mathbf {x}$ measures the proportion of pre-labeled objects in the same cluster as $\mathbf {x}$ that share the same class label as $\mathbf {x}$, including $\mathbf {x}$ itself. $\text {B}^{3}$ Recall measures the proportion of objects with the same class label as $\mathbf {x}$ sharing the same cluster with $\mathbf {x}$.

**Fig. 7:**
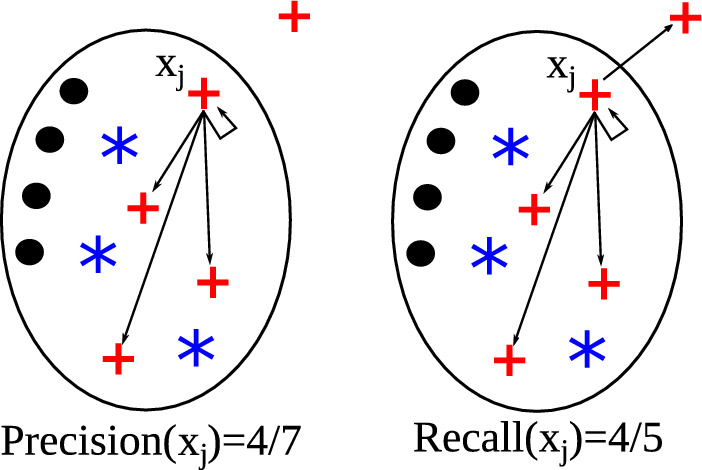
$\text {B}^{3}$ Precision and $\text {B}^{3}$ Recall of an object $\mathbf {x}_{j}$. Objects within the ellipse belong to the same cluster. Red crosses and blue stars represent two different class labels. The example assumes that the whole set of pre-labeled objects ($\mathbf {X}_L$, required to compute $B^3$ Recall) is displayed. Unlabeled objects are represented by black filled circles. Notice that any two objects with the same class label in the same cluster have the same precision and recall (Color figure online)

To compute $\text {B}^{3}$ Precision and $\text {B}^{3}$ Recall we consider only the set of pre-labeled objects. Given an object $\mathbf {x} \in \mathbf {X}_{L}$ with class label ${{\,\mathrm{class}\,}}(\mathbf {x})$ and a cluster $\mathbf {C}_{i}$ containing this object, the $\text {B}^{3}$ Precision of $\mathbf {x} \in \mathbf {C}_{i}$ can be formally defined as:

2 $$\begin{aligned} P_{B^{3}}(\mathbf {x}, \mathbf {C}_{i})= \dfrac{|\{\mathbf {x}'~|~\mathbf {x}' \in \{\mathbf {C}_{i} \cap \mathbf {X}_{L}\} \wedge {{\,\mathrm{class}\,}}(\mathbf {x})={{\,\mathrm{class}\,}}(\mathbf {x}') \}|}{|\{~\mathbf {x}'~|~\mathbf {x}' \in \{ \mathbf {C}_{i} \cap \mathbf {X}_{L}\}~\}|} \end{aligned}$$

and the $\text {B}^{3}$ Recall of $\mathbf {x} \in \mathbf {C}_{i}$ can be defined as:

3 $$\begin{aligned} R_{B^{3}}(\mathbf {x}, \mathbf {C}_{i})= \dfrac{|\{ \mathbf {x}'~|~\mathbf {x}' \in \{\mathbf {C}_{i} \cap \mathbf {X}_{L}\} \wedge {{\,\mathrm{class}\,}}(\mathbf {x})={{\,\mathrm{class}\,}}(\mathbf {x}') \}|}{|\{\mathbf {x}'~|~\mathbf {x}' \in \mathbf {X}_{L} \wedge {{\,\mathrm{class}\,}}(\mathbf {x})={{\,\mathrm{class}\,}}(\mathbf {x}') \}|} \end{aligned}$$

Figure 7 illustrates these concepts for a pre-labeled object $\mathbf {x}_{j}$ as part of a cluster with eleven objects, seven of them pre-labeled, four of which share the same label as $\mathbf {x}_{j}$. Obviously any two objects with the same class label in the same cluster share the very same values of $P_{B^{3}}$ and $R_{B^{3}}$, which allows fast computation.

As in traditional assessment of supervised classifiers, precision and recall capture two different aspects of an outcome, one of which will be overlooked if a single criterion is chosen. Just like the well-known F1-Measure in classification, a single conservative index (hereafter called $\text {B}^{3}$*F-Measure*) can be obtained by combining $P_{B^{3}}$ and $R_{B^{3}}$ taking their harmonic mean:

4 $$\begin{aligned} F_{B^{3}}(\mathbf {x}, \mathbf {C}_{i}) = \frac{2 P_{B^{3}}(\mathbf {x}, \mathbf {C}_{i}) \cdot R_{B^{3}}(\mathbf {x}, \mathbf {C}_{i})}{P_{B^{3}}(\mathbf {x}, \mathbf {C}_{i}) + R_{B^{3}}(\mathbf {x}, \mathbf {C}_{i})} \end{aligned}$$

The $\text {B}^{3}$ F-Measure in Eq. (4) can be used as a building block for an optimization criterion to extract clusters from the HDBSCAN* hierarchy using FOSC. Specifically, let $\{\mathbf {C}_{1}, \ldots , \mathbf {C}_{k}\}$ be the set of all candidate clusters in a HDBSCAN* hierarchy of $\mathbf {X}$ from which we want to extract a flat clustering solution in a semi-supervised way. The goal is to maximize the *Overall*$\text {B}^{3}$*F-Measure* of the resulting solution, which is an average $\text {B}^{3}$ F-Measure over the pre-labeled objects as belonging to their respective selected clusters:

5 $$\begin{aligned} Overall_{F_{B^{3}}} = \frac{1}{|\mathbf {X}_{L}|} \sum _{i=1}^{k} \left( \sum _{\mathbf {x} \in \{\mathbf {C}_{i} \cap \mathbf {X}_{L}\}} \delta _i \cdot F_{B^{3}}(\mathbf {x}, \mathbf {C}_{i}) \right) \end{aligned}$$

where $\delta _i$ is the indicator function used by FOSC to determine whether or not ($\delta _i = 1$ or $\delta _i = 0$, respectively) cluster $\mathbf {C}_{i}$ is selected to be part of the optimal flat solution.

Notice that the Overall $\text {B}^{3}$ F-Measure in Eq. (5) satisfies the properties of *additivity* and*locality* required by FOSC. In fact, it can be easily decomposed as:

6 $$\begin{aligned} Overall_{F_{B^{3}}} = \sum _{i=1}^{k} \delta _i \cdot \omega (\mathbf {C}_{i}) \end{aligned}$$

where

7 $$\begin{aligned} \omega (\mathbf {C}_{i}) = \frac{1}{|\mathbf {X}_{L}|} \left( \underset{\mathbf {x} \in \{\mathbf {C}_{i} \cap \mathbf {X}_{L}\}}{\sum } F_{B^{3}}(\mathbf {x}, \mathbf {C}_{i}) \right) \end{aligned}$$

The Overall $\text {B}^{3}$ F-Measure is therefore a sum of individual components $\omega (\mathbf {C}_{i})$ that can be pre-computed independently for each candidate cluster in the cluster tree as it depends solely on the information of the pre-labeled objects that belong to that cluster.

One could analogously define an *Overall*${B}^{3}$*Precision* and *Overall*${B}^{3}$*Recall* if desired, which could also be decomposed in the same way. We omit the details here as they follow closely and straightforwardly the development shown above.

Figure 6b, c, and d show the cluster tree for the hierarchy in Table 1 with the quality value of each cluster individually assessed by the decomposed components of the *Overall*${B}^{3}$*Precision*, *Overall*${B}^{3}$*Recall*, and *Overall*${B}^{3}$*F-Measure* as described above, respectively, for a collection of 5 pre-labeled objects, $\mathbf {X}_{L} = \{ \mathbf {x}_{1}, \mathbf {x}_{6}, \mathbf {x}_{8}, \mathbf {x}_{15}, \mathbf {x}_{18}\}$, with 4 different class labels (red cross, green hash, magenta triangle, and blue star).

The $\text {B}^{3}$ Precision, $\text {B}^{3}$ Recall, and $\text {B}^{3}$ F-Measure for singleton nodes corresponding to noise objects that are not pre-labeled (omitted for the sake of clarity) are undefined, and they are set to zero so that these nodes do not affect computations. The singleton node descendant from $\mathbf {C}_{2}$ containing $\mathbf {x}_{1}$, which is pre-labeled (red cross), does affect computations in all semi-supervised scenarios, as shown in Fig. 6b, c, and d.

Figure 6b shows that $\text {B}^{3}$ Precision has guided FOSC to extract the leaf nodes, as the corresponding clusters tend to be purer in class labels. In contrast, $\text {B}^{3}$ Recall has guided FOSC to extract the root (Fig. 6c).^7^ This is not necessarily always the case, in particular because there may be ties between the values of precision or recall of a cluster and that of its sub-clusters. In the semi-supervised setting, the way FOSC resolves ties in the values of the objective function involving different candidate solutions in a subtree is by taking the solution that would be chosen in the unsupervised setting (Campello et al. 2013b). In HDBSCAN*, this means that ties in the semi-supervised cluster extraction are decided using Stability (Campello et al. 2015). Notice that this is particularly important when certain regions of the data, corresponding to entire subtrees of the cluster tree, are not represented by labels at all, so decisions inside those subtrees can only be made in an unsupervised way.

The $\text {B}^{3}$ F-Measure provides a conservative (pessimistic) compromise between precision and recall, which in this particular example has led FOSC to extract the same solution as $\text {B}^{3}$ Precision (see Fig. 6b). In general, however, the solution extracted with $\text {B}^{3}$ F-Measure does not need to coincide with either $\text {B}^{3}$ Precision or $\text {B}^{3}$ Recall.

FOSC also allows cluster extraction by using an objective function that combines the unsupervised and semi-supervised measures into a single function. In particular, it has been shown in the original FOSC publication (Campello et al. 2015) that any convex combination of objective functions that satisfy the properties of additivity and locality required by the algorithm will also satisfy the property of additivity and locality, and its decomposition into local components corresponding to each individual cluster is given by the convex combination of the corresponding local components of the original objective functions.

Here, we experiment with the average between Overall Stability (unsupervised) and Overall $B^3$ F-Measure (semi-supervised). Since the scales of these different objective functions are different, they have to be normalized first. The $B^3$ F-Measure ranges within [0, 1], but Stability has no upper bound. In order to make the mixed measure commensurable in this case, the Stability of individual clusters can be divided by the overall value of Stability of the optimal flat solution that is extracted in the unsupervised case (Campello et al. 2013b). For example, recall that the optimal unsupervised solution in Fig. 6a has an Overall Stability of 14.95. We then divide the Stability of each cluster by this value before taking the average with the corresponding value of F-Measure. In our example, the result is shown in Fig. 6e.

Notice in Fig. 6e that, by combining both unsupervised and semi-supervised measures, FOSC has been able to extract a solution that is different from either case ($\mathbf {P} = \{\mathbf {C}_{4}, \mathbf {C}_{5}, \mathbf {C}_{3}\}$). In particular, since the Stability of $\mathbf {C}_{2}$ is not much higher than that of its sub-clusters combined, the presence of pre-labeled objects with different labels in that cluster has driven FOSC to choose the sub-clusters instead. Contrarily, in spite of the presence of pre-labeled objects with different labels in $\mathbf {C}_{3}$, FOSC has opted to keep this cluster rather than splitting it, because Stability strongly suggests that this is a single cluster. Of course, if full priority is to be given to user-defined labels, there is no point in using the mixed approach, and $B^3$ F-Measure should be used instead (Stability being only used to decide ties).

HDBSCAN* and FOSC take the so-called *soft* approach to semi-supervised clustering (Basu et al. 2008), in which labels or constraints are (prior) user expectations rather than hard constraints that must be enforced. The particular approach can be interpreted as a strategy that gives priority to satisfy the implicit model assumptions (density connectivity in the case of HDBSCAN*) when constructing the cluster hierarchy, and then use external information provided by the user as preferences (rather than hard requirements) to extract a flat solution (Campello et al. 2013b, 2015).

### Complexity

For a given cluster tree containing *k* candidate clusters, each of which has an associated quality measure that has been pre-computed, FOSC can be implemented in a very efficient way with two traversals through the cluster tree, one bottom-up as previously described and another one top-down just materializing the provisional selections. This means that the complexity of the algorithm is *O*(*k*), i.e., linear w.r.t. the number of nodes in the tree, both in terms of running time and memory space (Campello et al. 2013b). In simplified cluster trees such as those produced by HDBSCAN*, *k* is typically much smaller than the number of data objects ($k\ll n$). Even in an unlikely scenario where a binary cluster split is observed at each of the *n* (maximum) possible hierarchical levels, it follows that $k = 2n-1$ and, therefore, FOSC is *O*(*n*) in the worst-case (given a cluster tree and the corresponding values of cluster quality).

The unsupervised measure of Stability can be computed by HDBSCAN* “on-the-fly”, i.e., as the hierarchical levels are iteratively computed, so Stability does not increase the computational complexity of HDBSCAN*, which is $O(n^2)$ w.r.t. runtime,^8^ except for a small constant factor. Likewise, it should be clear that $B^3$ Precision, $B^3$ Recall, and $B^3$ F-Measure can be trivially computed for all candidate clusters simultaneously to the construction of the hierarchy, by just keeping track of the number of pre-labeled objects of each class in each cluster, which again can be done without affecting the computational complexity of HDBSCAN*. Even in the scenario where $B^3$ Precision, $B^3$ Recall, and $B^3$ F-Measure are computed afterwards, as a post-processing of the HDBSCAN* hierarchy, it is straightforward to compute these measures for each cluster with a single pass through the hierarchical levels for each pre-labeled object, updating the counts of the respective class label at each cluster the object belongs to in the cluster tree. In this case, the additional post-processing cost in the worst-case, where the clustering hierarchy has*n* levels, is $O(n \cdot |\mathbf {X}_L|)$, which again, does not change the computational complexity of HDBSCAN* since $|\mathbf {X}_L| < n$ (typically $|\mathbf {X}_L| \ll n$).

## Experimental setup

In this section we describe the experimental setup for the assessment of our proposed density-based methods for semi-supervised classification and for semi-supervised clustering, both in terms of effectiveness as well as in terms of computational efficiency. In Sect. 6.1 we describe the experimental setup for the classification scenario, which refers to the methods discussed in Sect. 4. In Sect. 6.2 we describe the setup for the clustering scenario, which refers to the methods described in Sect. 5. For the sake of reproducibility, all our codes are made publicly available in Github.^9^

### Semi-supervised classification

#### Performance measure

We report the macro-averaged F-measure, i.e., the average over all classes of the harmonic mean between precision and recall for each class (Sokolova and Lapalme 2009). We compute the F-measure based only on those objects whose labels have not been exposed to the semi-supervised classification algorithms for training.

**Table 2:** List of real datasets collected to perform the semi-supervised classification experiments

Dataset	#obj	#att	#cl	Distance
ACE ECFP4 (Sutherland et al. 2004)	114	1025	2	Tanimoto
ACE ECFP6 (Sutherland et al. 2004)	114	1025	2	Tanimoto
Analcatdata authorship (Vanschoren et al. 2013)	841	70	4	Cosine
Armstrong-v1 (de Souto et al. 2008)	72	1082	2	Cosine
Auto price (Vanschoren et al. 2013)	159	16	2	Euclidean
Bank note–Authentication (Vanschoren et al. 2013)	1372	5	2	Euclidean
Cardiotocography (Vanschoren et al. 2013)	2126	36	10	Euclidean
Chowdary (de Souto et al. 2008)	104	183	2	Cosine
Chcase Geyser1 (Vanschoren et al. 2013)	222	2	2	Euclidean
COX2 ECFP6 (Sutherland et al. 2004)	322	1025	2	Tanimoto
DHFR ECFP4 (Sutherland et al. 2004)	397	1025	2	Tanimoto
DHFR ECFP6 (Sutherland et al. 2004)	397	1025	2	Tanimoto
Diggle table (Vanschoren et al. 2013)	310	8	9	Euclidean
Fontaine ECFP4 (Fontaine et al. 2005)	435	1024	2	Tanimoto
Fontaine ECFP6 (Fontaine et al. 2005)	435	1024	2	Tanimoto
Gordon (de Souto et al. 2008)	181	1627	2	Cosine
Iris (Lichman 2013)	150	5	3	Euclidean
M1 ECFP4 (Gaulton et al. 2017)	769	1025	2	Tanimoto
M1 ECFP6 (Gaulton et al. 2017)	769	1025	2	Tanimoto
Mfeat-factors (Vanschoren et al. 2013)	2000	216	10	Euclidean
Mfeat-Karhunen (Vanschoren et al. 2013)	2000	65	10	Euclidean
Seeds (Lichman 2013)	210	8	3	Euclidean
Segmentation (Vanschoren et al. 2013)	2100	20	7	Euclidean
Semeion (Vanschoren et al. 2013)	1593	256	10	Cosine
Stock (Vanschoren et al. 2013)	950	10	2	Euclidean
Transplant (Vanschoren et al. 2013)	131	4	2	Euclidean
WDBC (Lichman 2013)	569	32	2	Euclidean
Wine (Lichman 2013)	178	13	3	Euclidean
Yeast galactose (Yeung et al. 2003)	205	81	4	Euclidean

#### Datasets

We use datasets with different characteristics (such as number of objects, number of attributes, and number of classes) and from different domains (biology, text, and broadly from the UCI machine learning repository (Lichman 2013)), requiring different distance measures, as summarized in Table 2.

Some datasets required pre-processing. The datasets “ACE” (ACE ECFP4 and ACE ECFP6), “COX2 ECPF6”, “DHFR” (DHFR ECFP4 and DHFR ECFP6), “Fontaine” (Fontaine ECFP4 and Fontaine ECFP6) and “M1” (M1 ECFP4 and M1 ECFP6) describe molecules utilized in the process of identifying relationships between chemical structure and biological activity (Rivera-Borroto et al. 2011). We transformed the data with *generatemd* (a tool in the JChem framework, available at http://www.chemaxon.com) into a set of binary attributes using two different configurations of the Extended-Connectivity Fingerprints (ECFP), with the maximum diameter of the circular neighbors considered for each atom set to 4 and 6, resulting in ECFP4 and ECFP6, respectively. We applied the Tanimoto dissimilarity to perform the experiments in the new set of attributes. For the datasets “Auto price”, “Bank note–Authentication”, “Stock”, and “Transplant” we used the Euclidean distance on the *z*-score normalized data objects, which relates to Pearson correlation in the original data space.

#### Pre-labeled data subsets

For the semi-supervision, we selected labeled objects from the datasets randomly, ensuring that there is at least one label from each class, repeating the random selection 30 times, thus resulting in 30 variants of each dataset for each percentage of labeled objects. To study the influence of the amount of labeled data, we take different percentages of labeled objects: $2\%$, $5\%$, $8\%$, and $10\%$.

#### Algorithms and parameters

The original implementation of HISSCLU has been provided by the authors. As further competitors we included three semi-supervised classification approaches: the Gaussian Field and Harmonic Function (GFHF) (Zhu et al. 2003), the Robust Multi-class Graph Transduction (RMGT) (Liu and Chang 2009), and the Laplacian Support Vector Machine (LapSVM) (Belkin et al. 2006), for which implementations are available from a former comparative study (de Sousa et al. 2013). Following the recommendations in that study, we construct a graph and weight matrix for the label propagation in these algorithms as follows (de Sousa et al. 2013):

1. Compute the distance matrix using the dissimilarity function listed in Table 2;
2. Construct the graph using a symmetric version of the *k*-nearest neighbors graph. The mutual *k*-nearest neighbors graph creates an edge between objects $\mathbf {x}_{i}$ and $\mathbf {x}_{j}$ if and only if they are one of the*k* closest neighbors of each other.
3. Compute the weight matrix applying the radial basis function kernel (RBF kernel):
  $$\begin{aligned} K(\mathbf {x}_{i}, \mathbf {x}_{j})= \exp \left( \frac{-d(\mathbf {x}_{i}, \mathbf {x}_{j})^{2}}{2\sigma ^{2}}\right) , \end{aligned}$$
  where $\sigma$ is the kernel bandwidth parameter.

We set the bandwidth parameter as $\sigma = \frac{\sum _{i=1}^n d\left( \mathbf {x}_{i}, \mathbf {x}_{i_k}\right) }{3n}$, following de Sousa et al. (2013), where $d(\mathbf {x}_{i}, \mathbf {x}_{i_k})$ denotes the distance between $\mathbf {x}_{i}$ and its *k*th nearest neighbor $\mathbf {x}_{i_k}$.

For the label propagation with algorithms GFHF and LapSVM in datasets with multiple classes, we use the standard *one-vs-all* combination of the binary classifiers.

For HISSCLU, HDBSCAN*(cd,wPWD), HDBSCAN*(cd,wMST), HDBSCAN*(ap,wPWD), and HDBSCAN*(ap,wMST) we set the parameters of the weighting function (to improve the separation between classes) as $\rho =50.0$ and $\xi =5.0$, which were also used in the original HISSCLU publication (Böhm and Plant 2008). For HDBSCAN*(ap,-), no parameter is required.

For the neighborhood size, we tested a smaller ($m_{\text {pts}}=k=4$) and a larger choice ($m_{\text {pts}}=k=15$). The density-based semi-supervised classification algorithms achieve better results on average with the smaller value (confirming observations in previous studies (Campello et al. 2015; Lelis and Sander 2009; Böhm and Plant 2008)), while GFHF, RMGT, and LapSVM show better performance on average with larger neighborhood size (also confirming previous findings (de Sousa et al. 2013)). Hence, to not give an unfair advantage to a class of algorithms, we run each of the algorithms with the value that works better for them on average, $m_{\text {pts}}=4$ for the density-based methods, and $k=15$ for the other ones.

#### Statistical test for performance comparison

To analyze the overall results, we applied the two-step procedure described by Demšar (2006). First, we applied the Friedman test (Friedman 1937) to examine whether there is a significant difference between the results of the algorithms on collected datasets. If the null hypothesis, assuming no significant difference between the algorithms, is rejected at the given p-value, the Nemenyi posthoc test (Nemenyi 1963) is applied to reveal which pairs of algorithms exhibit such differences. This test states that the performance of two different algorithms is significantly different if the corresponding average ranks differ by at least a Critical Difference (CD) value. In both tests, Friedman test and Nemenyi posthoc procedure, we selected a significance level of 5% ($\alpha =0.05$).

#### Runtime experiments

The datasets for the runtime experiment were generated randomly using “animals.c” (Lichman 2013). The program generates objects that have 72 attributes and that belong to one of 4 classes. We generated 20,000 instances for this dataset, and extracted subsets of size 100, 500, 1000, 5000, 10,000, and 15,000 instances, with an approximately equal distribution of the classes. We select randomly 30 labeled objects, at least one for each class, repeat the procedure 20 times, and present the mean runtime. We use the same parameters as in the effectiveness experiments for all algorithms.

### Semi-supervised clustering

#### Performance measures

In the semi-supervised clustering scenario, we report the results of our experiments using the Adjusted Rand Index (ARI) (Hubert and Arabie 1985), which is a standard external validation measure in the clustering literature (Jain and Dubes 1988). The index has been computed here *not* using any of the objects belonging to the set of pre-labeled objects. Noise objects are treated as singletons for the ARI computations.

#### Datasets

For controlled experiments where we know the true probability distribution of clusters, we use the benchmark collection of Handl and Knowles (2007),^10^ which contains 160 synthetic datasets with Gaussian and Ellipsoidal clusters. There are two sub-collections, one containing 80 low dimensional datasets (2D and 10D) and the other containing 80 high dimensional datasets (50D and 100D). The datasets have 4, 10, 20, and 40 clusters with cluster sizes varying from 10 to 500 depending on the number of clusters. For each combination of dimensionality and number of clusters there are 10 different datasets. We used Euclidean distance with this collection.

We also use real datasets from different domains. In particular, part of the datasets considered in the semi-supervised classification scenario (Sect. 6.1) are reused here in the semi-supervised clustering scenario. However, since classes as represented by classification labels do not necessarily correspond to clusters from a density-based perspective, we select only a subset of the datasets in Table 2 for which at least one clustering algorithm (amongst our proposed methods or amongst our baseline competitors) is able to achieve a solution with ARI of at least 0.50. This way, we ensure that the comparisons involve datasets for which it is possible to recover at least partially a clustering structure. The selected datasets are listed in Table 3.

**Table 3:** List of datasets collected to perform the semi-supervised clustering experiments

Dataset	#obj	#att	#cl	Distance
*Real*				
Articles-1442-5 Naldi et al. (2011)	253	4636	5	Cosine
Articles-1442-80 (Naldi et al. 2011)	253	388	5	Cosine
Bank note–Authentication (Vanschoren et al. 2013)	1372	5	2	Euclidean
Cardiotocography (Vanschoren et al. 2013)	2126	36	10	Euclidean
CellCycle-237 (Yeung et al. 2001)	237	17	4	Euclidean
Chowdary (de Souto et al. 2008)	104	183	2	Cosine
Diggle table (Vanschoren et al. 2013)	310	8	9	Euclidean
Ecoli (Lichman 2013)	336	7	8	Euclidean
Gordon (de Souto et al. 2008)	181	1627	2	Cosine
Iris (Lichman 2013)	150	5	3	Euclidean
Mfeat-factors (Vanschoren et al. 2013)	2000	216	10	Euclidean
Mfeat-Karhunen (Vanschoren et al. 2013)	2000	65	10	Euclidean
Seeds (Lichman 2013)	210	8	3	Euclidean
Segmentation (Vanschoren et al. 2013)	2100	20	7	Euclidean
Stock (Vanschoren et al. 2013)	950	10	2	Euclidean
WDBC (Lichman 2013)	569	32	2	Euclidean
Wine (Lichman 2013)	178	13	3	Euclidean
Yeast galactose (Yeung et al. 2003)	205	81	4	Euclidean
*ALOI collections*				
ALOI PCA (Horta and Campello 2012)	[50, 125]	6	[2, 5]	Euclidean
ALOI 88 (Horta and Campello 2012)	[50, 125]	88	[2, 5]	Euclidean
*Artificial collections*				
Gaussian (Handl and Knowles 2007)	[200, 5000]	[2, 10]	[4, 40]	Euclidean
Ellipsoid (Handl and Knowles 2007)	[200, 5000]	[50, 100]	[4, 40]	Euclidean

For the sake of completeness, we also include an additional dataset collection as well as additional individual datasets that have been used for clustering experiments in the HDBSCAN* paper (Campello et al. 2015), some of which are not included in Table 2. Specifically, datasets “Articles-1442-5” and “Articles-1442-80” consist of high dimensional (Bag-of-Words) representations of text documents, originally used by Naldi et al. (2011). These datasets are formed by 253 articles from 5 categories each, represented by 4636 and 388 dimensions, respectively. We used Cosine similarity for these datasets, as common in text analysis. Dataset “CellCycle-237” (Yeung et al. 2001) contains the expression levels of 237 genes, belonging to 4 known categories, across 17 conditions/dimensions. For this dataset we used Euclidean distance with *z*-score normalization of objects, which relates to Pearson correlation in the original data space (the standard measure in gene-expression analysis). Dataset “Ecoli” is from the UCI Repository (Lichman 2013), and contains 336 objects and 7 dimensions, with 8 classes. For this dataset we used Euclidean distance.

Finally, the “ALOI” collections in Table 3 consist of real datasets composed of image features of images extracted from the Amsterdam Library of Object Images (ALOI) (Geusebroek et al. 2005) and processed as described by Horta and Campello (2012). These datasets were created by randomly selecting *c* ALOI image categories as class (cluster) labels, 100 times for each $c=2, 3, 4, 5$, then sampling (without replacement), each time, 25 images from each of the *c* selected categories, resulting in 400 sets, each of which contains 2, 3, 4, or 5 classes (clusters) and 50, 75, 100, or 125 images (objects). The images were represented using 6 different image descriptors, with 144, 88, 128, 5, 44, and 256 attributes, respectively. The datasets using the *texture statistics* descriptor are denoted by “ALOI-TS88”, whereas “ALOI-PCA” stands for the datasets with a 6-dimensional representation combining the first principal component extracted from each of the six descriptors, using PCA. For both configurations, we used Euclidean distance.

Note that the “real” datasets are individual datasets, while ALOI and Artificial are sets of datasets. We will show summary results for each of the three categories (i.e., the collection of “real” datasets, the ALOI collections, and the articial collections) separately.

#### Pre-labeled data subsets

In order to obtain the subset of pre-labeled objects, $\mathbf {X}_L$, we randomly selected labeled objects from the datasets following two different strategies: (a) *non-controlled random*, where we draw objects without replacement, but under no further constraints; and (b) *controlled random*, where we ensure that a certain fraction of the class labels will (or will not) be missing in $\mathbf {X}_L$.

In the first, non-controlled random setting, it is *possible* that certain class labels will not be represented in the resulting subset of pre-labeled objects, which represents applications of semi-supervised clustering involving unknown categories yet to be discovered. In this setting, we varied the percentage of pre-labeled objects as 0% (unsupervised case, as a baseline), 1%, 2% and 5% of the data.

For the second, controlled random setting, we enforce that a fraction of the *c* class labels in the ground truth will be missing in $\mathbf {X}_L$. We experiment and compare the results with no classes missing (by ensuring that at least one object from each class is selected), $\lceil \frac{c}{2} \rceil$ classes missing, and all *c* classes missing (unsupervised case). For the first two cases, we varied the percentage of pre-labeled objects as 1%, 2% and 5% of the data. The results reported are averages over 50 random selections of the pre-labeled objects, for each dataset.

Some of the algorithms used as baseline competitors in our experiments perform semi-supervised clustering based on instance-level constraints, rather than on labels. For these algorithms, we also produce a corresponding set of instance-level constraints from each subset $\mathbf {X}_L$ of pre-labeled objects randomly drawn in our experiments; all possible pairwise constraints that hold in $\mathbf {X}_L$ are generated as follows: (a) a should-link constraint is created for each pair of objects with the same label in $\mathbf {X}_L$; and (b) a should-not-link constraint is created for each pair of objects with different labels in $\mathbf {X}_L$. Note that using instance-level constraints requires special care for a fair evaluation setup to not use information in the evaluation that has been implicitly exposed to the learning procedure (Pourrajabi et al. 2014). We performed the evaluation accordingly.

#### Algorithms and parameters

We include again HISSCLU as a competitor using the code provided by the authors, but now making use of the clustering extraction procedure described as a post-processing routine in the original publication (Böhm and Plant 2008), called*k-cluster* extraction (see Sect. 3.3.3). We also include as competitor SSDBSCAN, using the original code provided by Lelis and Sander (2009). We compare their original code against our improved code, hereafter referred to as SSDBSCAN++, which takes advantage of our density-based framework and runs the algorithm on top of a single, pre-computed $\text {MST}_r$, in contrast to the original algorithm, which dynamically and partially builds minimum spanning trees multiple times. As for the core algorithm in our framework, HDBSCAN*, we compare our new clustering extraction strategies with the strategies from the original publication (Campello et al. 2015), namely: unsupervised FOSC extraction based on *stability*, semi-supervised FOSC extraction based on instance-level pairwise constraints (unsupervised stability used to decide ties only), and the mixed case involving a balanced combination of these two strategies. We will call these competitors HDBSCAN*(UN), HDBSCAN*(CON), and HDBSCAN*(MixCON), respectively. Our proposed, label-based counterparts to HDBSCAN*(CON) and HDBSCAN*(MixCON) are called hereafter HDBSCAN*(BC) and HDBSCAN*(MixBC), respectively. The former performs semi-supervised FOSC extraction using the $\text {B}^{3}$ F-Measure, with the unsupervised criterion of stability being used to decide ties only; the latter is the mixed case involving a balanced combination of these two criteria, as discussed in Sect. 5.2.

All these algorithms share as a common parameter the neighborhood size, $m_{\text {pts}}$, which has been set in the same way as previously described for the semi-supervised classification experiments (Sect. 6.1). As for the additional parameters required by HISSCLU, $\rho$ and $\xi$, the setting previously adopted for the semi-supervised classification experiments, which was particularly effective in stretching class boundaries in that scenario, has shown not to be as effective in the clustering scenario. For this reason, we performed preliminary experiments with several combinations of these two parameters within the ranges $\rho \in [1,50]$ and $\xi \in [0.5, 5.0]$, and selected $\rho = 10.0$ and $\xi = 5.0$ as the most effective choice for the clustering experiments. The only new parameter used here, the value of *k* in HISSCLU’s *k-cluster* extraction routine, was set as $k = 0.2$, which was also used by the authors in the original publication Böhm and Plant (2008).

#### Statistical test and runtime experiments

For performance comparisons in the clustering scenario, we apply the same statistical validation procedure as described in Sect. 6.1.5 for the classification scenario. Runtime experiments follow the same experimental design as described in Sect. 6.1.6.

## Results 

In this section we describe and analyze the results of our experiments in the semi-supervised classification (Sect. 7.1) and semi-supervised clustering (Sect. 7.2) scenarios.

### Semi-supervised classification results

**Fig. 8:**
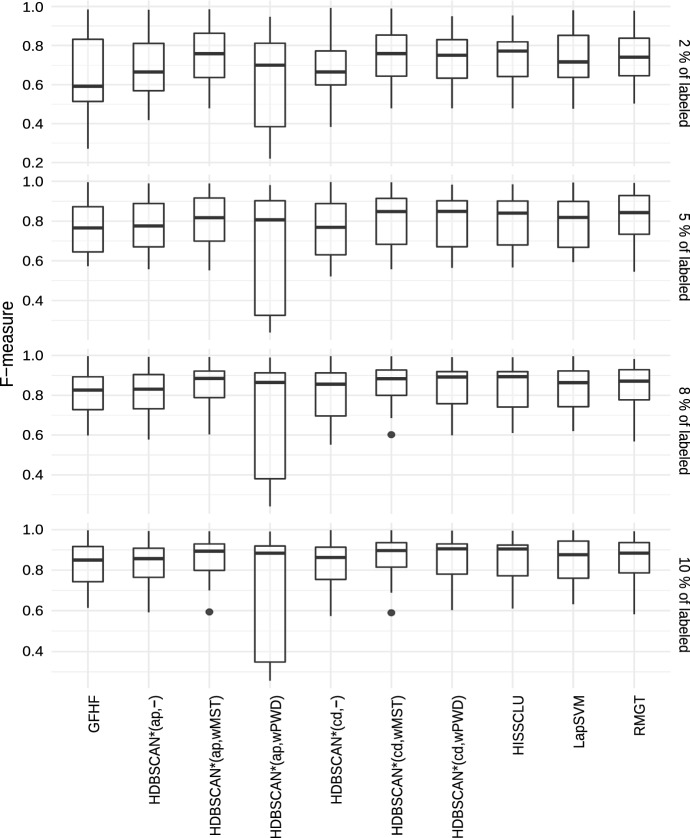
Quality of results obtained by the semi-supervised classification algorithms averaged over all datasets, separated by different percentage of labeled objects

#### Effectiveness

Figure 8 presents the distribution of the achieved quality over all datasets, separated by the percentage of labeled objects used for training. A straightforward and unsurprising observation is that all algorithms improve their performance with an increased amount of training data, and most show also a reduced variance of quality over the datasets. A noticeable exception to the reduced variance with larger amounts of training data is HDBSCAN*(ap,wPWD); its variance actually increases with more training data. A direct comparison with HDSBSCAN*(ap,wMST) and with HDBSCAN*(cd,wPWD) suggests that the combination of the all-points core distance and the label-based distance weighting applied to the distance matrix becomes more susceptible to random effects in the selection of labeled objects.

**Fig. 9:**
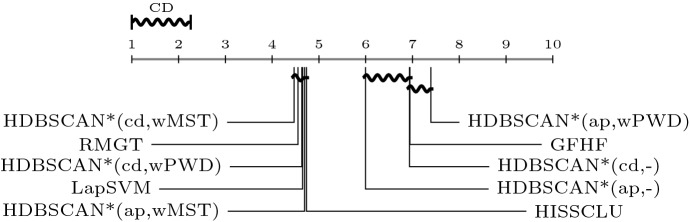
Average ranks and critical distance (CD) with statistical significance $\alpha =0.05$ according to the Friedman test

We see in general, that all variants have their strengths and weaknesses over the different datasets and dataset variants. When establishing a ranking of the algorithms and framework variants over the combined results (all datasets and all percentages of labeled objects), the Friedman and the Nemenyi post hoc tests check for statistical differences between the algorithms. Figure 9 visualizes the average ranks of the algorithms along with the critical distance. We can identify two distinct groups without statistical difference between the algorithms at confidence level $\alpha =0.05$.

The top group contains four density-based algorithms: HDBSCAN*(cd,wMST), HDBSCAN*(cd,wPWD), HDBSCAN*(ap,wMST), and HISSCLU, together with two traditional algorithms: RMGT and LapSVM. The critical distance indicates that the differences in ranking within this group are not significant. The lower group (consisting of two overlapping groups) comprises the HDBSCAN* variants without any label-based distance weighting, the variant HDBSCAN*(ap,wPWD) that we already observed as having a larger variance of quality, and the last of the traditional competitors, GFHF.

#### Efficiency

**Fig. 10:**
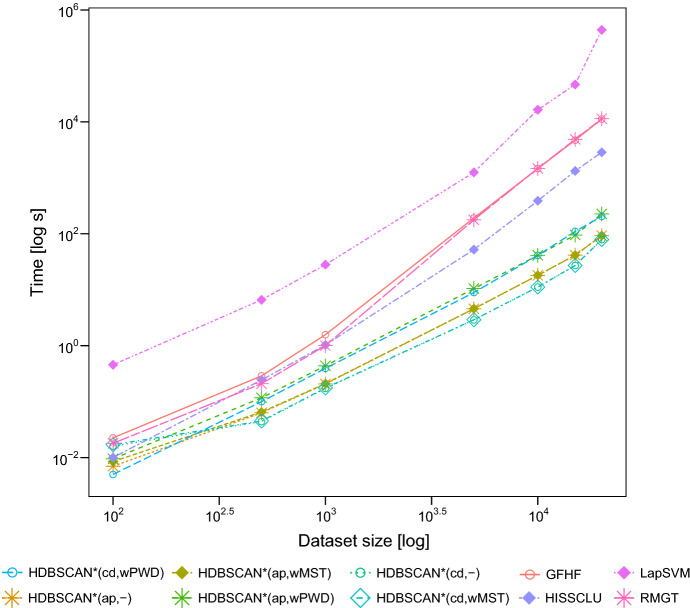
Runtime over dataset size (Color figure online)

Figure 10 depicts the runtime behavior of the tested algorithms when increasing the dataset size. Although comparisons of absolute runtime measurements are in our favor here, they should not be considered conclusive, especially when using different implementations (Kriegel et al. 2017). However, we can in fact observe that the algorithms LapSVM, GFHF, and RMGT have a worse runtime *behavior* compared to the density-based algorithms (note the log-log-scale of the plot). Among the density-based algorithms, HISSCLU is clearly worse than all the variants of our framework. Among our variants, applying weights on the complete distance matrix is again worse, as expected. Note that applying the weighting on the MST instead barely manifests itself in terms of runtime; compare HDBSCAN*(cd,-) versus HDBSCAN*(cd,wMST) and HDBSCAN*(ap,-) versus HDBSCAN*(ap,wMST): in both pairs the difference is hardly noticeable and is in the range of milliseconds. Applying weights on the distance matrix results a more substantial change.

#### Overall summary of findings (classification)

In brief, as a very objective take-home message learnt from our semi-supervised classification experiments, Figs. 8, 9 and 10 suggest that HDBSCAN*(cd,wMST) and HDBSCAN*(ap,wMST) possibly represent the best compromise between prediction power and computational cost, as these algorithms have exhibited top performance (Figs. 8, 9) in the representative collection of datasets used in our experiments, while appearing as two of the fastest algorithms according to the runtime evaluation in Fig. 10.

### Semi-supervised clustering results

#### Improved SSDBSCAN

We start by comparing the results of our improved implementation of SSDBSCAN, called SSDBSCAN++, against the results of the original code provided by Lelis and Sander (2009). The results averaged over each of the dataset collections in Table 3 are summarized in Table 4, for the non-controlled random scenario. The results in Table 4 show that, by using a single pre-computed $\text {MST}_r$, our improved implementation achieves at least the same quality of results as the original implementation, if not better (as highlighted in bold).

For the sake of compactness, we omit the results of the controlled random scenario, but the conclusions are the same. For this reason, we will include only SSDBSCAN++ in the subsequent experiments.

**Table 4:** ARI results (mean ± standard deviation) of the original SSDBSCAN implementation and our improved implementation, SSDBSCAN++, in the non-controlled random scenario with different amounts of pre-labeled objects

Collection	SSDBSCAN	SSDBSCAN++
*1% pre-labeled obj.*		
Real	$0.37 \pm 0.08$	$\mathbf {0.39 \pm 0.09}$
ALOI	$0.05 \pm 0.01$	$0.05 \pm 0.01$
Artificial	$0.63 \pm 0.01$	$\mathbf {0.66 \pm 0.01}$
*2% pre-labeled obj.*		
Real	$0.45 \pm 0.09$	$\mathbf {0.49 \pm 0.09}$
ALOI	$0.22 \pm 0.01$	$0.22 \pm 0.01$
Artificial	$0.73 \pm 0.01$	$\mathbf {0.77 \pm 0.01}$
*5% pre-labeled obj.*		
Real	$0.53 \pm 0.08$	$\mathbf {0.59 \pm 0.07}$
ALOI	$0.58 \pm 0.01$	$\mathbf {0.59 \pm 0.01}$
Artificial	$0.80 \pm 0.02$	$\mathbf {0.84 \pm 0.01}$

#### Effectiveness

**Fig. 11:**
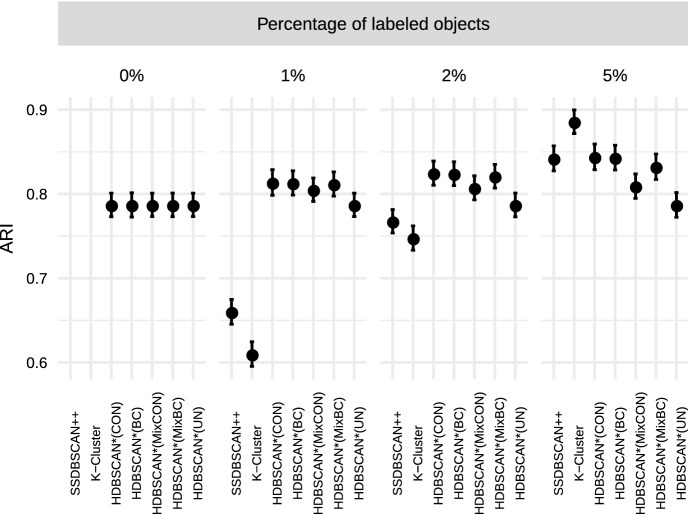
ARI results for the Artificial collection (non-controlled random scenario). Error bars denote 95% confidence intervals for the mean of means within each dataset

Figure 11 shows the summarized results of the compared algorithms for the “Artificial” collection, under the non-controlled random setup, separated by the percentage of labeled objects used for training. The first column (0%) corresponds to the unsupervised case, which emphasizes two facts: (a) neither SSDBSCAN nor HISSCLU’s *k*-cluster can operate in the absence of pre-labeled objects; and (b) in this scenario, all HDBSCAN* semi-supervised variants, which resolve ties in an unsupervised way, reduce to the unsupervised case (i.e., HDBSCAN*(UN)). As we move across the other columns from 1 to 5%, it is clear that all semi-supervised algorithms benefit from larger amounts of pre-labeled objects. However, unlike the semi-supervised variants of HDBSCAN*, which operate reliably all across the board, SSDBSCAN and *k*-cluster are unstable and provide competitive results only when using 5% of pre-labeled objects. When comparing our proposed methods, HDBSCAN*(BC) and HDBSCAN*(MixBC), against their constraint-based counterparts, HDBSCAN*(CON) and HDBSCAN*(MixCON), respectively, it is clear that, while HDBSCAN*(BC) and HDBSCAN*(CON) produce very similar results, HDBSCAN*(MixBC) outperforms HDBSCAN*(MixCON) in all scenarios.

**Fig. 12:**
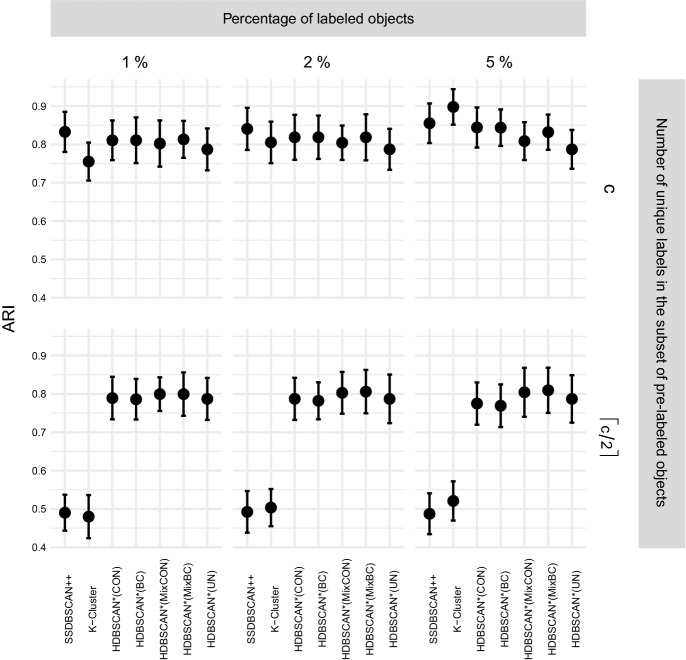
ARI results for the Artificial collection (controlled random scenario). Error bars denote 95% confidence intervals for the mean of means within each dataset

A possible reason for SSDBSCAN and HISSCLU’s *k*-cluster being unstable and producing poor results for smaller amounts of pre-labeled objects is that, in the non-controlled random experimental setup, there is no guarantee that all class labels are represented in the subset of pre-labeled objects, $\mathbf {X}_L$. To investigate this hypothesis, in Fig. 12 we show the results for the controlled random setup, where we contrast experiments where all *c* class labels in the ground truth are guaranteed to be represented in $\mathbf {X}_L$, against experiments with the same amount of pre-labeled objects but only approximately half of the *c* class labels allowed to be in $\mathbf {X}_L$. Notice that the extreme case in which no class label in the ground truth is represented in $\mathbf {X}_L$ corresponds to the unsupervised case, shown in the first column of Fig. 11.

Figure 12 confirms that, as expected, all algorithms are to some extent negatively affected by class labels missing in $\mathbf {X}_L$. However, while all the HDBSCAN* variants, including our proposed methods, are only slighted impacted, showing to be robust to this important factor in practical clustering applications, SSDBSCAN and*k*-cluster are strongly affected and show prominent drops in performance. Among the HDBSCAN* variants, it is noticeable that those variants that use the unsupervised criterion to decide ties only (i.e., HDBSCAN*(CON) and our HDBSCAN*(BC)), are slightly more sensitive to missing labels than the mixed variants, HDBSCAN*(MixCON) and our HDBSCAN*(MixBC), which instead combine the semi-supervised and the unsupervised criteria. For this reason, while HDBSCAN*(CON) and our HDBSCAN*(BC) perform slightly better than HDBSCAN*(MixCON) and our HDBSCAN*(MixBC) when no label in unrepresented, the opposite occurs when there are unrepresented labels.

**Fig. 13:**
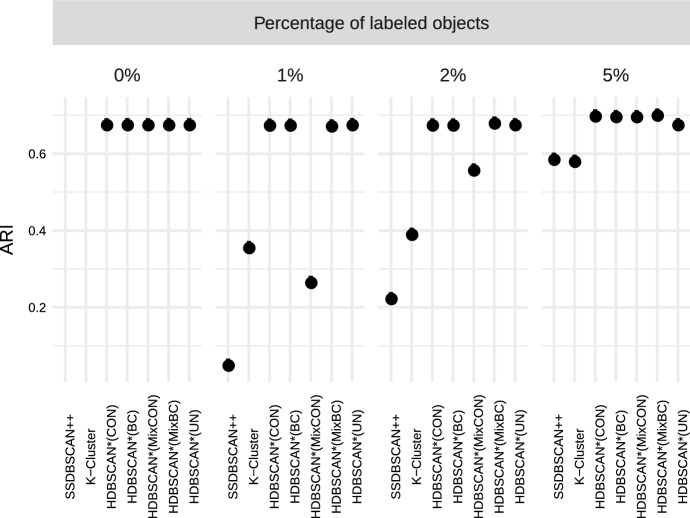
ARI results for the ALOI collection (non-controlled random scenario). Error bars denote 95% confidence intervals for the mean of means within each dataset

The results for the “ALOI” collection, under the non-controlled random experimental setup, are shown in Fig. 13. The conclusions that can be drawn are similar to those from the artificial collection (Fig. 11). The differences are: (a) semi-supervision improves only slightly the results in the ALOI datasets. In fact, the difference between the semi-supervised and the unsupervised results is only noticeable when 5% of pre-labeled objects are used; (b) there is a smaller variability in the average results across different datasets (error bars are almost indistinguishable); (c) SSDBSCAN and*k*-cluster have now underperformed in all scenarios, even when 5% of pre-labeled objects are used; and (d) this time, the constraint-based mixed variant, HDBSCAN*(MixCON), has also underperformed when only 1% or 2% of pre-labeled objects have been used.

**Fig. 14:**
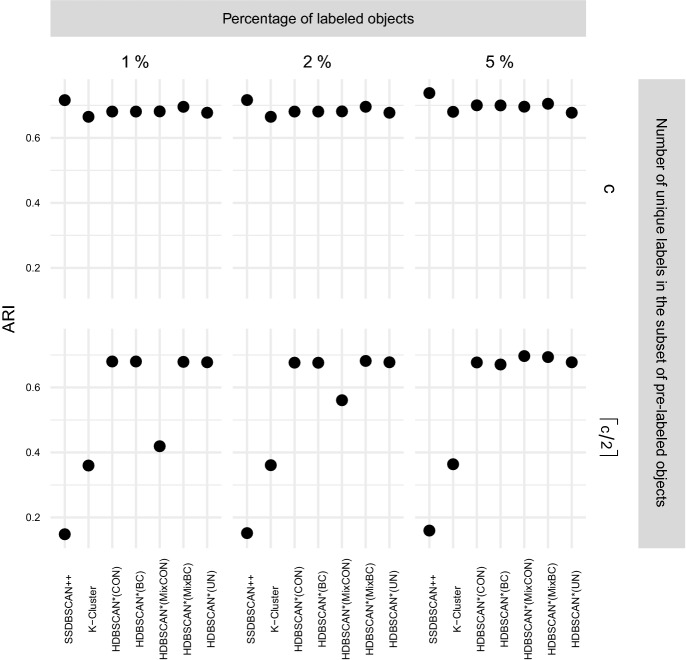
ARI results for the ALOI collection (controlled random scenario). Error bars denote 95% confidence intervals for the mean of means within each dataset

In order to investigate the cause for the underperformance of SSDBSCAN, *k*-cluster, and HDBSCAN*(MixCON), we show the results for the controlled random experiments in Fig. 14. While it is clear that the methods perform similarly when all class labels are represented in $\mathbf {X}_L$, with SSDBSCAN outstanding to some extent, the performances of SSDBSCAN, *k*-cluster, and HDBSCAN*(MixCON) (the latter w.r.t. the 1% and 2% scenarios only) drop substantially when there are categories not represented in $\mathbf {X}_L$.

**Fig. 15:**
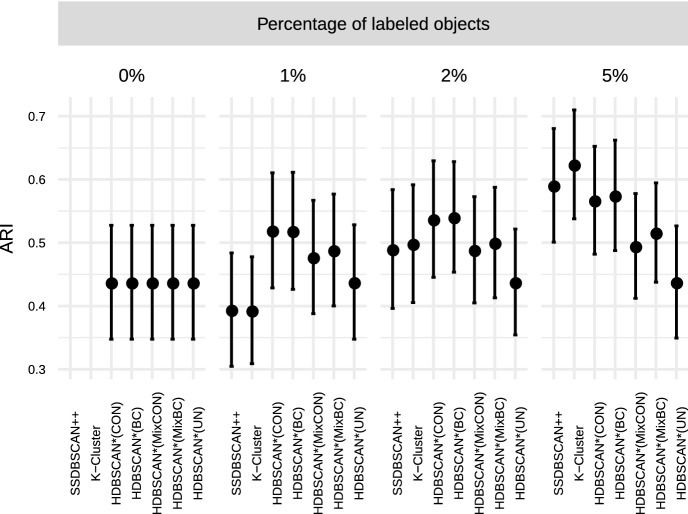
ARI results for the Real collection (non-controlled random scenario). Error bars denote 95% confidence intervals for the mean of means within each dataset

**Fig. 16:**
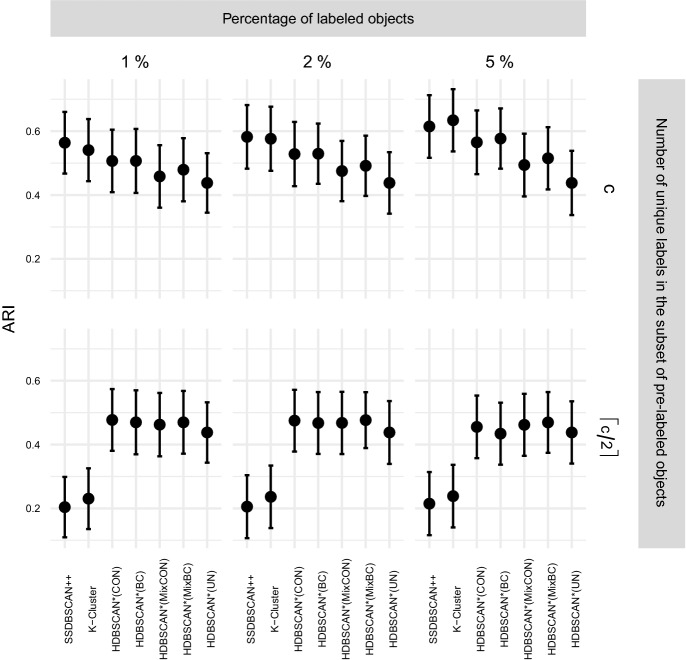
ARI results for the Real collection (controlled random scenario). Error bars denote 95% confidence intervals for the mean of means within each dataset

The results for the “Real” collection, under the non-controlled random experimental setup, are shown in Fig. 15. In this case, apart from the higher variability in the results (due to summarizing over very different datasets), the conclusions are essentially the same as those drawn from the artificial collection in Fig. 11. In particular, all semi-supervised algorithms benefit from larger amounts of pre-labeled objects, but while SSDBSCAN and *k*-cluster show superior results when using larger amounts of pre-labeled objects (5%), which increases the chances that all or most of the class labels are represented in $\mathbf {X}_L$, these two algorithms underperform when smaller amounts are used (1%). When comparing our label-based methods, HDBSCAN*(BC) and HDBSCAN*(MixBC), against their constraint-based counterparts, HDBSCAN*(CON) and HDBSCAN*(MixCON), respectively, the results are very similar, but slightly higher for the label-based variants. When comparing the mixed variants, HDBSCAN*(MixBC) and HDBSCAN*(MixCON), with their non-mixed counterparts, HDBSCAN*(BC) and HDBSCAN*(CON), respectively, the non-mixed variants, which use unsupervised evaluation only to decide ties, exhibit better results. However, looking at the results for the controlled random setup, in Fig. 16, it is clear that, similar to the artificial collection in Fig. 12, the negative impact of unrepresented labels is more noticeable for the non-mixed variants. Their drop in performance when changing from *c* (100%) to $\lceil c/2 \rceil$ (50%) represented labels is only less prominent than SSDBSCAN’s and *k*-cluster’s.

**Fig. 17:**
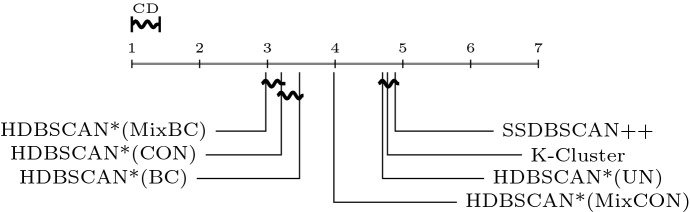
Average ranks and critical distance (CD) with statistical significance $\alpha =0.05$ according to the Friedman test: Artificial collection (non-controlled random scenario)

**Fig. 18:**
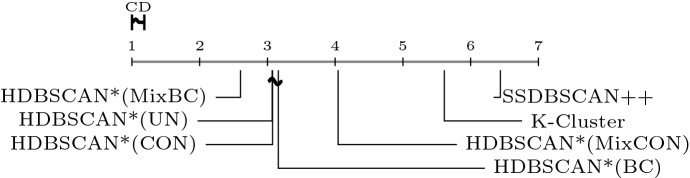
Average ranks and critical distance (CD) with statistical significance $\alpha =0.05$ according to the Friedman test: ALOI collection (non-controlled random scenario)

**Fig. 19:**
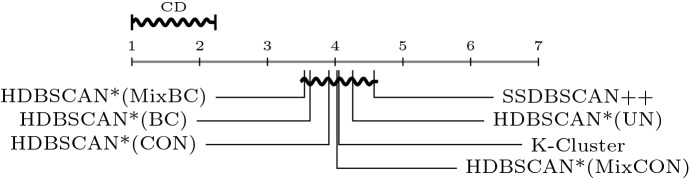
Average ranks and critical distance (CD) with statistical significance $\alpha =0.05$ according to the Friedman test: Real collection (non-controlled random scenario)

When establishing a ranking of the algorithms over the combined results of each collection separately (all datasets within the collection, all percentages of labeled objects, and 50 random subsets of pre-labeled objects for each combination of these), the Friedman and the Nemenyi post hoc tests check for statistical differences between the algorithms. Figures 17, 18, and 19 visualize the average ranks of the algorithms along with the critical distance of the test, for the collections Artificial, ALOI, and Real, respectively.

Some general conclusions based on the ranks are: (a) our proposed mixed variant, HDBSCAN*(MixBC), is the best ranked overall, in all three collections, with statistical difference to all other algorithms in the ALOI collection and to all algorithms but HDBSCAN*(CON) in the Artificial collection; and (b) when comparing our proposed HDBSCAN*(BC) with its constraint-based non-mixed counterpart, HDBSCAN*(CON), the latter is better ranked in the collections Artificial and ALOI, whereas the former is better ranked in the collection Real, but there is no statistical difference between these two variants in any of these cases.

#### Efficiency

**Fig. 20:**
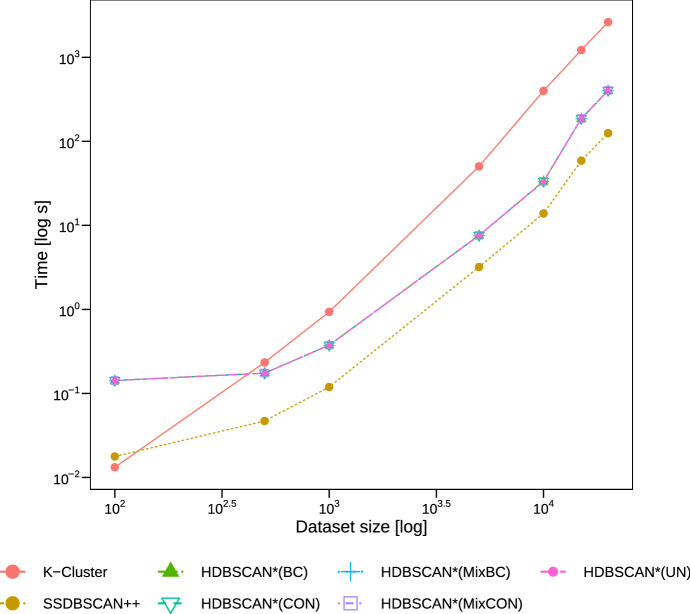
Runtime over dataset size (Color figure online)

Figure 20 depicts the runtime behavior of the tested algorithms. While SSDBSCAN runs the fastest in absolute terms, the *behavior* of the HDBSCAN* variants is similar, growing even slightly slower than SSDBSCAN in relative terms. In contrast, the runtime of HISSCLU’s *k*-cluster grows very noticeably at a much faster rate (note the log-log-scale). The different variants of HDBSCAN* are practically indistinguishable.

#### Overall summary of findings (clustering)

SSDBSCAN and HISSCLU’s *k*-cluster provide competitive results only for “larger” fractions of pre-labeled objects, where “large” has shown to be data-dependent and cannot be known in advance. Besides, these algorithms perform poorly unless all class labels are guaranteed to be represented in the subset of pre-labeled objects, which is a *classification* assumption and not an adequate assumption in the semi-supervised *clustering* scenario. If this assumption is met, then SSDBSCAN may be preferred as a much faster alternative to *k*-cluster, but in the general case we do not recommend either algorithm for semi-supervised*clustering* scenarios where there may be unknown classes yet to be discovered and, accordingly, not represented by any pre-labeled object. In these scenarios, we recommend one of the semi-supervised HDBSCAN* variants.

When comparing our proposed label-based variant HDBSCAN*(MixBC) with its existing constraint-based counterpart HDBSCAN*(MixCON) from Campello et al. (2015), our label-based version has systematically provided better results, and has the advantage of working directly with labels. When comparing our label-based non-mixed variant HDBSCAN*(BC) with its constraint-based counterpart HDBSCAN*(CON) from Campello et al. (2015), all our results suggest that the performances of these two methods are very close to each other, but the former has the advantage of working directly with labels. In summary, irrespective of whether a mixed or a non-mixed approach is used, there is no evidence to suggest that it is worth the additional effort of producing pairwise constraints from labels (when these are available), rather than working with labels directly as we propose.^11^ The mixed approach, in particular our proposed HDBSCAN*(MixBC), has shown to be more robust to unrepresented class labels, which led to a best rank performance overall when compared to all other algorithms.

## Conclusion

In this work, we first presented a unified view of density-based clustering algorithms that gives rise to a framework of semi-supervised density-based classification. We showed experimentally that several instances of the proposed framework can achieve comparable or better quality than HISSCLU and other traditional methods from the semi-supervised classification literature, namely RMGT, LapSVM, and GFHF, while being computationally more efficient as well as interpretable from a density-based, non-parametric viewpoint.

In addition, we extended HDBSCAN*, which plays a central role in our unified view and framework for density-based classification, to also perform semi-supervised clustering from a collection of pre-labeled data objects, rather than pairwise constraints (as previously supported by the algorithm). The direct use of labels has been shown to be both simpler and more effective. The results obtained in semi-supervised clustering scenarios where one or more categories in the data are not represented in the collection of pre-labeled objects are far superior than competitors from the literature, such as HISSCLU and SSDBSCAN.

In future work, a possible line of research in the context of semi-supervised classification is the use of the $\text {MST}_r$ graph (as opposed to traditional graphs such as mutual *k* nearest neighbors) in conjunction with other graph-based label propagation algorithms from the literature. In the context of clustering, an interesting topic for investigation is the problem of model selection in the semi-supervised scenario, namely, the direct use of labels rather than constraints to guide the choice of the $m_{\text {pts}}$ parameter in HDBSCAN*.

## Figures and Tables

**Fig. 21:**
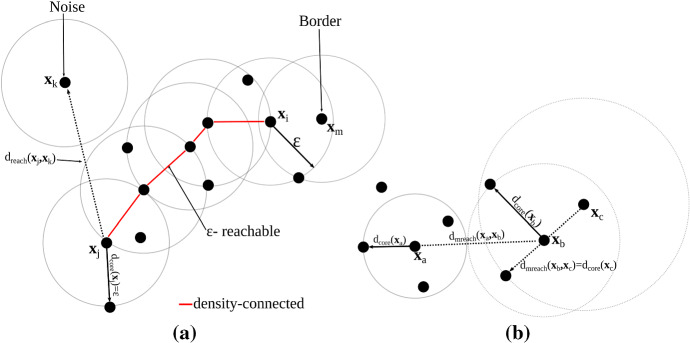
Graphical representation of fundamental density-based concepts and definitions ($m_{\text {pts}}=4$): **a** a noise and a border object, $\mathbf {x}_k$ and $\mathbf {x}_m$, are indicated for a given radius $\epsilon$. The asymmetric reachability distance between objects $\mathbf {x}_j$ and $\mathbf {x}_k$, $d_{\text {reach}}(\mathbf {x}_j, \mathbf {x}_k)$, which in this case coincides with the base distance between these objects, is also indicated. The concept of density-connectivity between two objects, $\mathbf {x}_i$ and $\mathbf {x}_j$, based on the definition of (direct or transitive) $\epsilon$-reachability between pairs of objects, is highlighted as a red line. **b** the core distances of objects $\mathbf {x}_a$, $\mathbf {x}_b$, and $\mathbf {x}_c$ are indicated. The symmetric (i.e., mutual) reachability distance between objects $\mathbf {x}_a$ and $\mathbf {x}_b$ is given by the base distance between these objects, whereas the mutual reachability distance between $\mathbf {x}_b$ and $\mathbf {x}_c$ is given by the core distance of $\mathbf {x}_c$ (Color figure online)

**Table 5:**
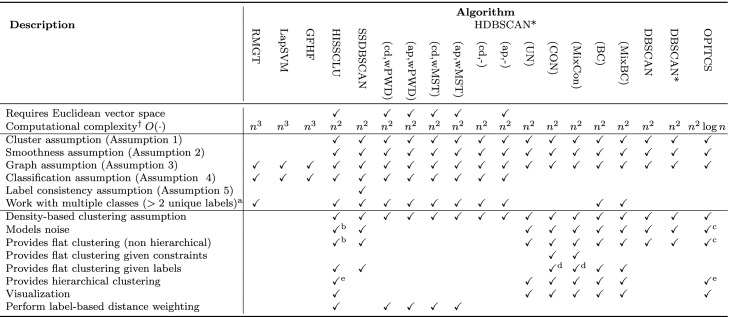
Summary of properties of the algorithms described in the document

The table consists of three blocks. The first block presents some general characteristics. The second block presents characteristics related to semi-supervised classification. The third block presents aspects related to clustering

$^{\mathrm{a}}$This property only applies to label-based semi-supervised algorithms;

$^{\mathrm{b}}$Optional k-cluster extraction procedure required;

$^{\mathrm{c}}$Optional cluster extraction procedure required;

$^{\mathrm{d}}$Notice that pairwise constraints can be produced from labels (but the opposite is not true);

$^{\mathrm{e}}$Implicitly encoded in a reachability plot only;

$^{\dag }$The given complexities are theoretical worst case complexities. With clever implementations (such as the use of suitable data structures), and many common application scenarios, the observed runtime of some of the algorithms can be significantly faster in practice

## Appendix

### Illustration of basic definitions

Figure 21 provides an illustration of the main concepts and definitions introduced in Sect. 3 and used throughout the paper, for readers less familiar with density-based data mining methods and algorithms.

### Summary of algorithms and their main properties

A summary of the properties and assumptions of all the density-based algorithms studied in this paper, including our new algorithms for semi-supervised classification and clustering introduced in Sects. 4 and 5, is provided in Table 5.

### Label expansion algorithm

Given a graph $\text {MST}_r$ pre-computed by HDBSCAN*, in our framework for semi-supervised classification (Sect. 4) we implement a label expansion method on top of the $\text {MST}_r$ to propagate the labels based on the path with the smallest largest edge to a labeled object, resolving ties, possibly consecutively, by considering the smaller of the next largest edge on the paths (Gertrudes et al. 2018).

This can be implemented by starting with a “connected component” $C_i$ for each pre-labeled object $\mathbf {x}_i \in \mathbf {X}_L$ that initially contains only $\mathbf {x}_i$. These connected components $C_i$ are then iteratively extended by traversing the $\text {MST}_r$ in the following way. The outgoing edges from each $C_i$ in the $\text {MST}_r$ are maintained in a sorted order, from smallest to largest edge weight. Then, always the smallest edge weight of all such edges (connecting to any of the $C_i$) is determined, and all of those edges with this edge weight are selected simultaneously. First, the selected edges that establish a connection between the current connected components will be considered: components that have the same label and are connected with selected edges will be merged into a single connected component that replaces them, whereas selected edges between connected components with different labels are ignored. Then, all objects $\mathbf {x}_o \in \mathbf {X}_U$ that connect to the selected edges and that have not been labeled yet will be considered in the following cases:

1. only a single edge, i.e., a single current connected component $C_i$ connects to an object $\mathbf {x}_o$; in this case, $\mathbf {x}_o$ and the corresponding edge are added to $C_i$ and ${{\,\mathrm{class}\,}}(\mathbf {x}_o)$ is set to ${{\,\mathrm{class}\,}}(C_i)$;
2. more than one edge, i.e., components $C_{i_1},\ldots , C_{i_m}$ connect to object $\mathbf {x}_o$; in this case, two sub-cases are possible:
  - all the components connected to $\mathbf {x}_o$ have the same label. In this sub-case all these components (including $\mathbf {x}_o$ and the corresponding edges) are merged into a single component;
  - the components connecting to $\mathbf {x}_o$ have different labels. In this sub-case, the algorithm determines the component $C_{i_j}$ that has the smallest largest edge (or, in case of ties, the smallest of the next largest edge, *etc*.) on a path to $\mathbf {x}_o$ from a pre-labeled object inside that component, $\mathbf {x}_o$ and its edge are added to $C_{i_j}$, ${{\,\mathrm{class}\,}}(\mathbf {x}_o)$ is set to ${{\,\mathrm{class}\,}}(C_{i_j})$, and all other components connecting to $\mathbf {x}_o$ that have the same class label as ${{\,\mathrm{class}\,}}(C_{i_j})$ are merged with $C_{i_j}$ (other components with different labels are not merged in this step, even if some of them share a label).

In both cases (a) and (b), the still unprocessed edges of the $\text {MST}_r$ incident to $\mathbf {x}_o$ (if any) will be added to the sorted set of outgoing edges from the current components, and the sorted set will be rearranged accordingly. If none of the selected edges in an iteration fit into one of the above scenarios, simply the next smallest edge weight from the sorted set is selected, and this process is repeated until all edges in the $\text {MST}_r$ have been processed (each edge will be processed once).^12^

The information of the smallest largest edge connecting a given unlabeled object $\mathbf {x}_o$ to one of the pre-labeled objects inside a given component $C_{i}$ can be efficiently stored in the objects themselves and expanded alongside with the labels. Let us suppose that an object $\mathbf {x}_o \in \mathbf {X}_U$ has just been added to component $C_{i}$ via an edge $(\mathbf {x}_o,\mathbf {x}_i)$ connecting $\mathbf {x}_o$ to $\mathbf {x}_i \in C_{i}$. Let $w_{o,i}$ be this edge’s weight, and let $w_{i}$ be the smallest largest edge weight connecting $\mathbf {x}_i$ to a pre-labeled object inside $C_{i}$ ($w_{i} = 0$ if $\mathbf {x}_i$ itself is pre-labeled, i.e., if $\mathbf {x}_i \in \mathbf {X}_L$). Then, the smallest largest edge weight on the path from $\mathbf {x}_o$ to a pre-labeled object inside $C_{i}$ can be stored in $\mathbf {x}_o$ as $w_o = \max \{w_{i},w_{o,i}\}$. These values will be stored in objects in the frontier of the components as these components grow. By doing this, the components $C_{i}$ do not need to be materialized, they will be implicitly encoded in the expanded labels.

Notice that, if an unlabeled object $\mathbf {x}_o$ is simultaneously connected to more than one component (cases (b)-i or (b)-ii above), say $C_i$ and $C_j$, via selected edges $(\mathbf {x}_o,\mathbf {x}_i)$ and $(\mathbf {x}_o,\mathbf {x}_j)$ with weights $w_{o,i} = w_{o,j}$, then the value $w_o$ to be stored in $\mathbf {x}_o$ will be the smaller of $\max \{w_{i},w_{o,i}\}$ and $\max \{w_{j},w_{o,j}\}$ (i.e., the smallest largest edge weight connecting $\mathbf {x}_o$ to one of the pre-labeled objects either inside $C_i$ or inside $C_j$). The smaller of these values will also decide the label of $\mathbf {x}_o$ in case ${{\,\mathrm{class}\,}}(C_i) \ne {{\,\mathrm{class}\,}}(C_j)$ (i.e., case (b)-ii). In order to solve ties ($\max \{w_{i},w_{o,i}\} = \max \{w_{j},w_{o,j}\}$), instead of keeping only the smallest largest edge weight stored in each object, we keep a sorted list of the *c* largest weights on the respective paths, where *c* is a constant. This way, we do not need to traverse the $\text {MST}_r$ to decide which component has the smallest next largest edge in case of ties. Only if *c* values are tied a traversal would be required. But this is unlikely in practice, and it would mean that the label is highly undecided, so breaking the tie randomly is nearly as accurate yet much faster. Since *c* is a constant (we use $c=5$ in our experiments), it does not affect the algorithm’s runtime or memory complexity.

A simplified pseudo-code corresponding to the aforementioned label expansion procedure is presented in Algorithms 1 and 2.

## Footnotes

1 Please refer to the “Appendix” for a graphical illustration of border objects as well as other fundamental definitions reviewed in this section, which will be subsequently used throughout the remainder of the manuscript.

2 Algorithmically, however, $\mathbf {x}_{3}$ may in practice be labeled depending on how the tie is resolved in the backtracking step of the label expansion procedure, and the final label may in this case be order-dependend.

3 HDBSCAN* is equipped with an *optional* parameter, $m_{\text {ClSize}}$, which allows the user to specify the minimum size for a component to be considered a cluster, in such a way that components with fewer than $m_{\text {ClSize}}$ objects are disregarded as noise. This can significantly reduce the size of the resulting clustering hierarchy. By default, HDBSCAN* uses $m_{\text {ClSize}} = m_{\text {pts}}$, so in practice only $m_{\text {pts}}$ needs to be given as input to the algorithm (Campello et al. 2013a, 2015). The result in Table 1 corresponds to $m_{\text {ClSize}} = m_{\text {pts}} = 3$.

4 There is no such thing as a “rag bag cluster” because noise objects should not be seen as clustered with each other, in spite of sharing a common label “0”.

5 Both properties could be seen as related to the “locality” property of *clustering functions* as introduced by Ackerman et al. (2010).

6 It also makes it possible to compute Stability for the root, $\mathbf {C}_1$.

7 The root can be trivially removed from the set of candidate solutions in FOSC, if desired (Campello et al. 2013b).

8 It can be implemented in *O*(*n*) w.r.t. memory (Campello et al. 2015).

9 https://github.com/jadsoncastro/UnifiedView/wiki.

10 Available at http://personalpages.manchester.ac.uk/staff/Julia.Handl/generators.html.

11 Please notice that this statement holds under the assumption that labels are available. In certain application scenarios users will only be able or willing to provide pairwise constraints, and we are by no means claiming that these constraints cannot be useful for clustering. The relevance of pairwise constraints in semi-supervised clustering has been thoroughly investigated in the literature—e.g. see (Lampert et al. 2018) for a recent study in the realm of time-series.
